# Supplementary material for: Protocol for a prospective, observational, deep phenotyping study on adipose epigenetic and lipidomic determinants of metabolic homoeostasis in South Asian Indians: the Indian Diabetes and Metabolic Health (InDiMeT) study
Source: BMJ Open. 2021 May 6;11(5):e043644. doi: 10.1136/bmjopen-2020-043644 (PMC8103950; doi:10.1136/bmjopen-2020-043644)
Supplement: Author's manuscript [file bmjopen-2020-043644.draft_revisions.pdf]

BMJ Open is committed to open peer review. As part of this commitment we make the peer review history of every article we publish publicly available.

When an article is published we post the peer reviewers' comments and the authors' responses online. We also post the versions of the paper that were used during peer review. These are the versions that the peer review comments apply to.

The versions of the paper that follow are the versions that were submitted during the peer review process. They are not the versions of record or the final published versions. They should not be cited or distributed as the published version of this manuscript.

BMJ Open is an open access journal and the full, final, typeset and author-corrected version of record of the manuscript is available on our site with no access controls, subscription charges or pay-per-view fees (http://bmjopen.bmj.com).

If you have any questions on BMJ Open's open peer review process please email info.bmjopen@bmj.com

BMJ Open

Protocol for a prospective, observational, deep phenotyping study on adipose epigenetic and lipidomic determinants of metabolic homeostasis in South Asian Indians: The Indian Diabetes and Metabolic Health (InDiMeT) Study

Journal:	*BMJ Open*
Manuscript ID	bmjopen-2020-043644
Article Type:	Protocol
Date Submitted by the Author:	10-Aug-2020

Complete List of Authors:	Nadiger, Nikhil; St John's National Academy of Health Sciences, Division of Nutrition<br>Devi, Sarita; St John's National Academy of Health Sciences, Division of Nutrition<br>Thomas, Tinku; St John's Medical College Hospital, Division of Epidemiology and Biostatistics<br>Sivadas, Ambily; St John's National Academy of Health Sciences, Division of Nutrition<br>Raj-Kuriyan, Rebecca; St John's National Academy of Health Sciences, Division of Nutrition<br>Govindaraj, Sridar; St John's Medical College Hospital, General Surgery<br>Kurpad, Anura; St John's National Academy of Health Sciences, Division of Nutrition<br>mukhopadhyay, arpita; St John's National Academy of Health Sciences, Division of Nutrition

Keywords:	DIABETES & ENDOCRINOLOGY, Physiology < NATURAL SCIENCE DISCIPLINES, MOLECULAR BIOLOGY, Biochemistry < NATURAL SCIENCE DISCIPLINES, GENETICS

SCHOLARONE™
Manuscripts

I, the Submitting Author has the right to grant and does grant on behalf of all authors of the Work (as defined in the below author licence), an exclusive licence and/or a non-exclusive licence for contributions from authors who are: i) UK Crown employees; ii) where BMJ has agreed a CC-BY licence shall apply, and/or iii) in accordance with the terms applicable for US Federal Government officers or employees acting as part of their official duties; on a worldwide, perpetual, irrevocable, royalty-free basis to BMJ Publishing Group Ltd ("BMJ") its licensees and where the relevant Journal is co-owned by BMJ to the co-owners of the Journal, to publish the Work in this journal and any other BMJ products and to exploit all rights, as set out in our licence.

The Submitting Author accepts and understands that any supply made under these terms is made by BMJ to the Submitting Author unless you are acting as an employee on behalf of your employer or a postgraduate student of an affiliated institution which is paying any applicable article publishing charge ("APC") for Open Access articles. Where the Submitting Author wishes to make the Work available on an Open Access basis (and intends to pay the relevant APC), the terms of reuse of such Open Access shall be governed by a Creative Commons licence – details of these licences and which Creative Commons licence will apply to this Work are set out in our licence referred to above.

Other than as permitted in any relevant BMJ Author's Self Archiving Policies, I confirm this Work has not been accepted for publication elsewhere, is not being considered for publication elsewhere and does not duplicate material already published. I confirm all authors consent to publication of this Work and authorise the granting of this licence.

For peer review only

Protocol for a prospective, observational, deep phenotyping study on adipose epigenetic and lipidomic determinants of metabolic homeostasis in South Asian Indians: The Indian Diabetes and Metabolic Health (InDiMeT) Study

Nikhil Nadiger[1], Sarita Devi[1], Tinku Thomas[2], Ambily Sivadas[1], Rebecca Raj-Kuriyan[1], Sridar Govindaraj[3], Anura V. Kurpad[1] and Arpita Mukhopadhyay[1, *]

[1]Division of Nutrition, St John's Research Institute, Bangalore, India

[2]Department of Biostatistics, St. John's Medical College and Hospital, Bangalore, India

[3]Department of General Surgery, St John's Medical College and Hospital, Bangalore, India.

Corresponding author:

Division of Nutrition,

St John's Research Institute,

Sarjapur Road, Bangalore, India. PIN: 560034

Phone: +91-80-49467000

Fax: +91-80-25501088

Email address: arpitam@sjri.res.in

Word count: 3379

For peer review only

ABSTRACT

Introduction

We describe the rationale and broad study design of the InDiMeT study, a new prospective study incorporating extensive epigenetic and lipidomic signatures to examine their association with the dysregulation of adipose de novo lipogenesis (DNL) in South Asian Indians. The InDiMeT study aims to use a case-control design to identify genetic and modifiable-environmental-lifestyle associated determinants of a) epigenomic dysregulation of adipose DNL in type 2 diabetes mellitus (T2DM) adipose tissue, b) identify correlates of epigenomic dysregulation of adipose DNL in peripheral blood mononuclear cells (PBMCs) from T2DM subjects and c) elucidate plasma lipidomic correlates of adipose DNL in T2DM that can be used as biomarkers of adipose tissue dysfunction.

Methods and analysis

The InDiMeT study will involve recruitment of 176 normoglycaemic and T2DM individuals who will be undergoing laparoscopic surgery for clinical conditions. Extensive phenotyping of the subjects will be conducted and epigenetics and lipidomic measurements will be made. The adipose DNL pathway genes are likely to be hypermethylated in T2DM patients with corresponding reduction of gene expression. Correlates of epigenomic dysregulation of adipose DNL pathway in PBMCs and their adipose and plasma lipidomic signatures in T2DM subjects could act as early markers of development of T2DM.

Ethics and Dissemination

For the InDiMeT study, ethical approval for addressing the specific aims has been applied from the Institutional Ethics Committee, St. John's Medical College and Hospital, St. John's National Academy of Health Sciences, Bangalore. Findings from this study will be disseminated through scientific publications in peer-reviewed journals, research conferences

For peer review only

and via presentations to stakeholders, patients, clinicians, public and policymakers through appropriate channels.

Keywords: type 2 diabetes mellitus, South Asian Indians, genotyping, metabolome, lipidome, pathophysiology, adipose tissue, de novo lipogenesis

For peer review only

ARTICLE SUMMARY

Strengths and limitations of this study

- The InDiMeT study will assess the epigenome-lipidome connection in relation to their contribution to adipose de novo lipogenesis (DNL) and metabolic homeostasis.
- The prospective case-control study design will permit us to assess the blood epigenetic and lipidomic markers that exhibit correlations with adipose DNL dysregulation can also potentially serve as minimally-invasive early biomarkers of dysglycemia.
- Understanding the epigenetic mechanisms and their associated lipidomic profiles related to dysregulated adipose DNL in South Asian Indians will provide the rationale for effective interventional strategies targeted to this population and discriminative diagnostic/prognostic biomarkers for prevention of T2DM in this high-risk population.
- The small sample size of 176 individuals is a likely limitation of the study.
- The subjects being recruited are the individuals who come for treatment of their preexisting clinical conditions and could not be a random representation of the total population.

INTRODUCTION

India is experiencing a type 2 diabetes mellitus (T2DM) epidemic, attributed to rapid sociodemographic and nutritional changes.[1] Younger, non-obese South Asian Indians develop insulin resistance, attributed to inappropriately elevated fat accretion.[2–4] An important reason for elevated/ectopic fat deposition in South Asian Indians may be their uniquely high carbohydrate diets (~65 energy%), which has been associated with elevated risk of T2DM, likely due to induction of hepatic insulin resistance through increased hepatic de novo lipogenesis (DNL, **Figure 1**).[5–7]

For peer review only

Unlike hepatic DNL, adipose DNL is understood to be associated with preservation of insulin sensitivity and an overall favourable metabolic phenotype ('metabotype').[8] Strawford et al reported negative correlations between adipose DNL and plasma glucose in humans.[9] Mice overexpressing adipose-specific GLUT4 are obese but paradoxically insulin sensitive, likely mediated by elevated adipocyte glucose influx-induced DNL.[10] Further, adipose-specific GLUT4 overexpression can rescue muscle-specific GLUT4 knockout-mediated insulin resistance, in a background of increased adiposity.[11] Therefore, it can be posited that white adipose tissue (AT) versus hepatic DNL may have opposed effects with respect to obesity-associated metabolic complications. Thus, understanding the contribution of adipose DNL in healthy versus T2DM-associated obesity is critical.

Understanding the epigenetic mechanisms and their associated lipidome profiles related to exacerbated adipose DNL in South Asian Indians will provide the rationale for effective interventional strategies targeted to this population and discriminative diagnostic/prognostic biomarkers for prevention of T2DM in this high-risk population.

Rationale and Specific Aims

The overarching goal of the Indian Diabetes and Metabolic Health (InDiMeT) study is to identify etiologically valuable determinants that affect susceptibility to type 2 diabetes at individual, sub-group and population level by testing the hypothesis that the epigenetic dysregulation of adipose DNL induced by high carbohydrate diet and environmental factors is an important step in the development adipose tissue dysfunction leading to type 2 diabetes (**Figure 2**) in India. The InDiMeT study proposes to achieve this by conducting detailed phenotyping and phenome measurements in a prospective manner in a high-risk population setting. Though animal studies have underlined the epigenetic changes in the adipose associated, few have investigated adipose epigenetics in humans and none in the context of

For peer review only

adipose DNL dysregulation and T2DM [12–15] which will help to identify epigenomic dysregulation of adipose DNL in T2DM AT. Although DNA methylation signatures are specific to tissue and cell types, only ~38% of the DNA methylation probes have been reported to be differentially methylated in blood in a tissue-specific fashion, implying likelihood of the rest ~62% probes being conservatively methylated between blood and other tissues.[16] Since blood is an easily available tissue, correlates of epigenomic dysregulation of adipose DNL in peripheral blood mononuclear cells (PBMCs) from T2DM subjects will be evaluated to validate the potential use of PBMCs as surrogates of AT dysfunction.

The overall outline of the InDiMeT study is depicted in **Figure 3**. The specific aims of this study are:

1. To identify epigenomic dysregulation of adipose de novo lipogenesis (DNL) in T2DM adipose tissue
2. To identify correlates of epigenomic dysregulation of adipose DNL in PBMCs from T2DM subjects
3. To elucidate plasma lipidomic correlates of adipose DNL in T2DM that can be used as biomarkers of adipose tissue dysfunction

METHODS AND ANALYSIS

Study setting

The InDiMeT study will be conducted in a tertiary care hospital in Bangalore district, India. It is surrounded by the state of Tamil Nadu in the southeast, the Bangalore Rural district in the northeast and northwest, and the Ramanagara district in the southwest.[17] As per the Indian 2011 Census, the Bangalore district recorded a decadal growth rate increase of 12% in the decade of 2001-2011 compared to the previous decade. This growth was ~4 times higher in the urban areas compared to the rural ones. The sex ratio of the Bangalore district was 916,

For peer review only

an 8-point increase compared to the 2001 Census. The literacy rate was 88% while the work participation rate was 44%, higher for males at 62% compared to females at 25%. As per the National Family Health Survey-4, one-third of the women and 26% of the men in Bangalore (Bangalore urban district) are overweight or obese.[18] In Bangalore urban district, prevalence of elevated random blood sugar measurements stands at 8.3% amongst women and 10.9% amongst men which compares to Karnataka state-wide figures of 6.3% for women and 8.4% for men respectively (**Figure 4**).

Study design and population

The InDiMeT study is designed as a prospective tertiary care hospital-based cohort study. 176 normoglycemic and T2DM individuals will be recruited into the study after explanation of details of the study and obtaining informed consent at the department of General surgery, St. John's Medical College and Hospital, St. John's National Academy of Health Sciences, Bangalore. Dissemination of information about the broader study goals and likely benefits to the community as well as engagement of potential subjects from the hospital will be initiated in August 2020. Recruitment will commence in September 2020 and continue till the recruitment target (176 individuals) is achieved. Strategies elaborated below will be employed for recruitment:

- Chart reviews – From the existing group of patients coming in to the Department of General Surgery, St. John's Medical College and Hospital, subjects are screened and then contacted to request for their participation.
- Databases – From the database of patients coming to the St. John's Medical College and Hospital, those that are eligible to participate in the study are contacted for possible recruitment.

For peer review only

Inclusion criteria: 35-60 years age; normoglycaemic and T2DM; normal and high BMI; men and women who are undergoing laparoscopic surgeries for their clinical conditions such as cholecystectomies, hernia or splenectomy will be invited to participate in the study.

Exclusion criteria: Age outside the range of 35-60 years, individuals who are unwilling to participate in the study, individuals participating in any other study and those who tested positive for hepatitis (HBsAg), HIV or syphilis (VDRL) infections. Those who have serious pre-existing medical condition, those undergoing laparoscopic surgery for clinical conditions other than cholecystectomies, hernia or splenectomy will be excluded, and these will be defined as conditions that require chronic or daily medical therapy, including medications, for T2DM, connective tissue diseases, inflammatory bowel disease, active tuberculosis and symptomatic heart disease.

Sample size calculation: Based on the study by Nilsson et al, the sample size required to observe a mean difference of 7% in DNA methylation in AT with an SD of 10% between T2DM and normoglycaemic controls with 90% power and 5% level of significance is 44 per group. As AT DNA methylation differences could be associated with BMI of the subjects, 44 individuals will be recruited in the normal (BMI<25 kg/m^2) and high BMI (BMI≥25 kg/m^2) groups of normoglycaemic controls and T2DM (35-60 years, men and women).[12]

For peer review only

Data collection

Socio-demographic data collection

Detailed socio-demographic data will be collected by trained personnel using well-structured questionnaires as elaborated in **Table 1**. The questionnaire will be provided and explained in English or local language in which the subject is most comfortable.

Socioeconomic status will be calculated based on scoring relevant for the Indian scenario.[19]

Table 1. Exposure and outcome measurements within the InDiMeT Study.

**General questionnaires**
Socio-demographic
Age, ethnicity and religion, family composition, education, employment status and type, household income, socioeconomic status
Medical history
Medical history related to birth weight, chronic and infectious diseases, last one month's morbidity conditions, current and past usage of tobacco (cigarettes, beedis or chewing tobacco) and alcohol, family history of diabetes and cardiovascular disease, details on most recent routine clinical investigations (haemoglobin, fasting/random/post-prandial glucose, OGTT glucose, HbA1C, serum insulin and C-peptide, urea, creatinine, Calcium, Phosphorus, TSH, Uric acid, serum cholesterol, HDL, LDL, VLDL, triglycerides, serum amylase and lipase, total protein, albumin, total bilirubin, direct and indirect bilirubin, SGOT, SGPT, alkaline phosphatase, GGT, sodium, potassium, chloride, routine urine test, urine albumin)
Medication and supplement use
Type, brand, dose and duration of medication, Vitamin and mineral supplements (clinician prescribed or over the counter)
Dietary habits
3 months food-frequency questionnaire
Physical activity
Daily, weekly and monthly frequency of sports activities, manual labour related hobbies and household chores, sedentary activities; occupational classification in terms of physical activity; sleep habits
**Anthropometry, body composition measurements and laboratory assessments**

For peer review only

Vital sign measurements	
Blood pressure, heart rate and pulse	
Body composition and anthropometry measurements	
DXA scanning, measurement of height and weight	
Laboratory assessments	
Blood investigations: Blood glucose level, Glycosylated Haemoglobin (HbA1C), serum insulin, C-peptide, C Reactive Protein (CRP), Glucagon-like peptide (GLP) and Glucose-dependent insulinotropic polypeptide (GIP)	
**Biobanking**	
Collection of blood samples	
Separation and storage of plasma for lipidomic analyses	
Collection of abdominal visceral adipose tissue	
Done during laparoscopic surgeries; collection and storage of adipose tissue for lipidomic, epigenomic, genomic and gene expression analyses	
**Phenome measurements**	
Non-targeted lipidomic analyses	
on adipose tissue and plasma samples	
Epigenomic analysis	
on adipose tissue samples and PBMCs	
Gene expression analysis	
on adipose tissue samples	
Genomic analysis	
on PBMCs	

OGTT: oral glucose tolerance test, TSH: thyroid-stimulating hormone, HDL: high-density lipoprotein, LDL: low-density lipoprotein, VLDL: very low-density lipoprotein, SGOT: serum

For peer review only

glutamic oxaloacetic transaminase, SGPT: serum glutamic pyruvic transaminase, GGT: gamma-glutamyl transferase, DXA: dual-energy X-ray absorptiometry, GAD: glutamic acid decarboxylase, PBMC: peripheral blood mononuclear cells

Medical history and medication and supplement use data collection

Participants will be asked regarding their birth weight, recent morbidity conditions and the medications and supplements they are currently taking. Details of the type and usage of these medications and supplements will be recorded by trained staff (Table 1). Participants will be requested to provide these details from the prescriptions or their hospital records will be sought after.

Vital sign measurements

Participants will be requested to relax and then blood pressure, heart rate and pulse will be measured and recorded by trained staff.

Body composition measurement by DXA scanning and anthropometry

Participants will be weighed in minimal clothing using a digital scale, to a precision of 0.1 kg. The height of the subjects will be recorded to the nearest 0.1 cm. Whole body and regional body composition will also be estimated using DXA (DPXMD 7254, Lunar Corporation, Madison, WI). The total body fat (BF) will be measured and both fat and muscle (ALST) will be expressed as a percentage of body weight (% BF and % ALST).

Laboratory assessments

Blood samples before and after their surgery will be collected from the participants. These samples will be utilized for assessments as elaborated in **Table 1**. Muscle insulin

For peer review only

sensitivity and β-cell function will be assessed by the homeostasis model assessment-of insulin resistance (HOMA-IR) and of β-cell function (HOMA-B) respectively.[20,21]

Lifestyle factors

Dietary data collection

The dietary intake will be assessed using a validated food frequency questionnaire (FFQ). The FFQ will be administered by trained personnel to obtain information about the habitual dietary intake for the preceding 3 months [22]. The nutrients and food groups will be estimated for all the foods listed in the FFQ and summed to obtain the total nutrient or food group intake per day for an individual. Nutrient information will be assessed for 27 macronutrients and micronutrients.

Physical activity data collection

The habitual physical activity and sleep habits of the subjects will be assessed by detailed and validated physical activity questionnaire.[23]

Biobanking

Blood collection and storage

Blood samples will be collected before and after the surgery by phlebotomy in EDTA/serum tubes, kept on ice, and immediately processed for aliquoting and storage at -80°C.

Abdominal visceral adipose tissue collection and storage

Intraabdominal omental adipose tissue will be collected during laparoscopic surgery with clinical indications of cholecystectomy, meshplasty (hernia) or splenectomy. The

For peer review only

collected tissue will be appropriately stored for phenome measurements (epigenomic and non-targeted lipidomic analyses) as described below.

Phenome measurements

Epigenomic analysis

Nucleic acids will be extracted from peripheral blood mononuclear cells (PBMCs) and adipose tissue. Epigenome-wide assessment of DNA methylation levels in CpG islands will be done by reduced representation bisulfite sequencing (RRBS) after bisulfite conversion.[12,24,25]

Gene expression by quantitative real-time PCR (qPCR) will be performed with SYBR Green chemistry for the top ten differentially methylated loci and for the adipose DNL pathway related genes: *MLXIPL, SREBP1, ACLY, FASN, CPT1A, CPT1B, SCD* and *ELOVL6*.

Genotyping of the subjects will be done using Metabochip (a custom genotyping array for metabolic and cardiovascular traits) or equivalent single nucleotide polymorphism (SNP) array, such as the Infinium Global Screening Array (Illumina Inc., USA).[26,27]

Non-targeted lipidomic analysis

Plasma and omental AT samples will be used for non-targeted lipidomic analysis. Samples will be spiked with SPLASH™ Lipidomix Mass Spec standard (Avanti Polar Lipids, USA) before lipid extraction and reconstitution. Non-targeted lipidomic analysis will be performed on a high-resolution analytical platform consisting a Vanquish Flex Binary UHPLC coupled to a Q Exactive Orbitrap, high-resolution accurate-mass spectrometer (LC-HRAM-MS, Thermo Scientific, USA). High resolution accurate mass data generated by Thermo Scientific Q Exactive Orbitrap will be identified and quantified by Thermo Scientific Lipid Search™ software. The following lipid species are of special interest: palmitic acid, stearic

For peer review only

acid, oleic acid, myristic acid, palmitoleic acid, acyl carnitines, palmitic acid esters of hydroxyl stearic acids (PAHSAs), furan fatty acid metabolite 3-carboxy-4-methyl-5-propyl-2-furanpropanoic acid (CMPF).[28–31]

Assessment of adipose DNL will be done by determining AT triglyceride (TG) content of the fatty acids myristic acid (14:0) and stearic acid (18:0) that have been reported to be correlated with plasma non-esterified fatty acids (NEFA) 14:0 and 18:0 but not with plasma NEFA 15:0, that is exclusively derived from dietary dairy sources.[32] Further, AT lipid species (from non-targeted lipidomic data) that positively correlate with transcript abundances of adipose DNL pathway genes will be identified as indicators of adipose DNL.

Plasma NEFA correlates of adipose DNL will be identified in the T2DM and normoglycaemic subjects by correlation analysis of plasma lipidomic data with adipose DNL measures [AT triglyceride (TG) content of the fatty acids myristic acid (14:0) and stearic acid (18:0)]. This is a novel strategy for estimating adipose DNL and identifying plasma correlates of adipose DNL as such indirect methods do not exist at present and the direct stable isotope based method of measuring DNL primarily measures post-prandial hepatic DNL rates.[33]

Data analysis plan

Continuous data will be presented as either mean ± SD or median (quartile1, quartile 3). The normality of distribution will be examined using the Shapiro-Wilk test and Q-Q plots. Demographic and clinical characteristics will be compared between cases of T2DM and controls by Student's t-test, if normally distributed or Mann-Whitney U test, if non-normally distributed, for continuous variables and by Chi-square test for categorical variables.

For analysis of RRBS data, the read sequences from the RRBS libraries will be adapted/quality trimmed using Trim Galore[34] and aligned to the reference human genome using a wildcard mapper like BSMap.[35] DNA methylation scores (β values) will be

For peer review only

calculated from the aligned reads.[36] Differentially methylated position (DMP) analysis will be conducted using limma software while adjusting for cell proportions (for PBMCs) and including age and sex as covariates.[37] Statistical significance will be set at $P<0.05$ following adjustment for multiple testing using the Benjamini Hochberg false discovery rate (FDR).[38] Differentially methylated regions (DMRs) will be defined as three or more contiguous probes within a 2 kb distance, each sharing the same direction of methylation change, each achieving a Benjamini Hochberg FDR corrected $P<0.05$ in DMP analysis. Enrichment analysis will be performed to understand the top significantly deregulated gene ontologies and pathways. Additionally, in-depth analysis of methylation status of the CpG islands in the promoter and coding regions of genes associated with the DNL pathway will be conducted.

Association studies between the CpG methylated regions and clinical traits will be performed using linear mixed-model package pyLMM and significant associations will be determined using Bonferroni corrected p-value cut-offs.

Gene expression values will be estimated by normalizing target gene expression against expression of housekeeping genes followed by comparison of target gene expression between the groups.

Methylation Quantitative Trait Loci (mQTLs) will be identified by testing for association between methylation levels and genotypes of the samples using FDR correction

Correlation-based clustering analysis of the lipid profiles will be performed and visualized as heatmaps to evaluate the interrelationships between the lipid species and for identifying signatures that differentiate between T2DM and control samples using supervised and unsupervised approaches. For heatmap analysis, only significantly altered features (significant alterations will be defined) will be analyzed and data will be organized using a Euclidean distance map and complete clustering algorithm. Random forest will be used to assess the classification performance of the lipidomics signatures. The significantly perturbed

For peer review only

pathways will be identified based on the differentially regulated lipids. For random forest and pathway impact analyses, data will be filtered using the interquartile range (IQR) function and scaled using auto-scaling, in which values are mean-centered and divided by the standard deviation of each variable. For the random forest analysis, the number of predictors to try for each node will be set to the square root of the total number of variables.

To identify the adipose lipid species that correlate with transcript abundances of genes in the adipose DNL pathway, first correlation analysis (Pearson's correlation, for normally distributed data and Spearman's correlation, for non-normally distributed data) will be conducted. This will be followed by regression analysis, with levels of lipid species exhibiting significant correlations as dependent variable and transcript abundance of individual genes in the adipose DNL pathway as independent variables, adjusting for probable confounders such as BMI and sex, if found to be significantly correlated with the dependent variables.

All statistical analysis will be processed and analyzed using a combination of SPSS Version 25 (IBM, USA), STATA 14 (StataCorp, USA) and R (R Foundation for Statistical Computing, Vienna).

Patient and public involvement

Patients and general public were not invited to comment on the study design and were not consulted to formulate study outcomes. Results for the InDiMeT study will be communicated to the patients and public in two complementary ways. Data related to individual participants of the study will be shared with them, if deemed beneficial for improving their health status, in consultation with clinician collaborators on this study and specific participant or their primary care providers. This is mandated by our Institutional Ethics Committee as well. The overall study results will be disseminated to participating individuals and public via appropriate forums for public science outreach.

For peer review only

ETHICS AND DISSEMINATION

Ethical considerations and informed consent

The objectives and aims of the study will be explained in subject's preferred local language to interested volunteers who fulfill the inclusion criteria. The Patient Information Sheet will contain relevant and specific information about the study, collection and storage of samples, genetic analysis and safe storage of subject data. Informed consent will be obtained from subjects before the start of the protocol.

Application for Institutional ethical approval has been submitted to the Institutional Ethics Committee, St. John's Medical College and Hospital, St. John's National Academy of Health Sciences, Bangalore. Subject recruitments will be initiated after the approval is received.

Data storage, management and protection

Data and resources generated as part of this study will be managed, stored and shared in line with Institutional, State and National policies. In brief, specifics are as follows:

- *Organization*: Conventions for naming and storage organization of data files as well as samples from human subjects (adipose and blood) will be set up, documented and circulated amongst study team members at the beginning of the study.
- *Storage*: Data will be stored in a secure manner in three copies in two different media. Back-up copies of data will be accompanied with metadata that describe the stored data in a standard, understandable format. Data storage and protection support will be obtained from the Institutional Information Technology team.
- *Physical samples*: Samples from the study subjects will be processed and stored as per the experimental protocols. Samples in excess of the requirements of the study

For peer review only

objectives will be stored appropriately as per the Institutional policies as well as policies of the peer-reviewed journals where findings from the study will be submitted for possible publications.

Dissemination of data

Sequence data generated from this study will be deposited in GenBank and epigenetic data in Gene Expression Omnibus, while raw and processed lipidomic data will be deposited in appropriate repositories such as Metabolomics Workbench or MetaboLights during the time of publication. Findings from the InDiMeT study will be appropriately communicated in refereed journals and forums for scientific and community science outreach.

SIGNIFICANCE AND OUTLOOK

The InDiMeT study will provide the base and act as a robust tool for mechanistic interpretations of public health and epidemiological aspects of developing T2DM and its complications in the Indian setting. Though a few large, prospective cohort studies to identify the determinants of T2DM are being conducted in India, to the best of our knowledge, none of them involve stated deep phenotyping or extensive phenome measurements of the participants.[39,40] This study was conceptualized to fill this niche in this country. The carefully monitored and quality-controlled collection and archiving of biological samples, along with availability of anthropometric, socio-demographic, nutritional and medical data on the participants will aid toward planning and implementation of an array of hypothesis-driven mechanistic studies.

The InDiMeT study will not be without limitations. Notable ones will include the low probability of participation by individuals of specific subsets of the population due to the voluntary nature of participation. The subjects being recruited are the individuals who come for treatment of their preexisting clinical conditions could not be a random representation of

For peer review only

the total population. Further, the study setting being a tertiary care hospital in a large, urban area in India, a country known for its large regional variations in cuisine and culture-associated dietary habits, composition and amounts of habitual dietary intakes of the study subjects are likely to be heterogenous.

Acknowledgements

The contribution of research fellow Ms. Bajanai Nongkhlaw, research assistant Ms. Beena Bose and nutritionist Mr. J. Jayakumar is gratefully acknowledged.

Authors' contributions

The authors of the manuscript and their individual contributions towards the manuscript are as follows. AM, TT and AVK: designed the research; NN, SD and SG: conduct of the study; AM, TT, AS, RR-K and AVK: analysis of the data, performing statistical analyses and interpretation of the results; AM and AVK: obtained funding; AM: had the idea for and led writing of the manuscript and had primary responsibility for the final content of the manuscript; and all authors critically reviewed the manuscript, read and approved the final version of the manuscript.

Funding

This study is supported by the Wellcome Trust/DBT India Alliance Fellowship awarded to AM [grant number IA/CPHI/19/1/504593] and to AVK [grant number IA/M/14/1/501681] and by the Department of Biotechnology, Government of India [Grant Sanction order BT/PR24342/PFN/20/1314/2017 to AM and AVK].

Competing interests

For peer review only

None declared.

For peer review only

References

1 World Health Organization. Global Health Estimates: Deaths by Cause, Age, Sex and Country, 2000-2012. 2014.

2 Misra A, Madhavan M, Vikram NK, *et al.* Simple anthropometric measures identify fasting hyperinsulinemia and clustering of cardiovascular risk factors in {Asian} {Indian} adolescents. *Metabolism* 2006;**55**:1569–73. doi:10.1016/j.metabol.2006.06.029

3 Frayling TM, Wiltshire S, Hitman GA, *et al.* Young-Onset Type 2 Diabetes Families Are the Major Contributors to Genetic Loci in the Diabetes UK Warren 2 Genome Scan and Identify Putative Novel Loci on Chromosomes 8q21, 21q22, and 22q11. *Diabetes* 2003;**52**:1857–63. doi:10.2337/diabetes.52.7.1857

4 Barker DJP. The Developmental Origins of Adult Disease. *J Am Coll Nutr* 2004;**23**:588S-595S. doi:10.1080/07315724.2004.10719428

5 Mohan V, Radhika G, Sathya RM, *et al.* Dietary carbohydrates, glycaemic load, food groups and newly detected type 2 diabetes among urban Asian Indian population in Chennai, India (Chennai Urban Rural Epidemiology Study 59). *Br J Nutr* 2009;**102**:1498–506. doi:10.1017/S0007114509990468

6 Acheson KJ, Schutz Y, Bessard T, *et al.* Carbohydrate metabolism and de novo lipogenesis in human obesity. *Am J Clin Nutr* 1987;**45**:78–85. doi:10.1093/ajcn/45.1.78

7 Perry RJ, Samuel VT, Petersen KF, *et al.* The role of hepatic lipids in hepatic insulin resistance and type 2 diabetes. *Nature* 2014;**510**:84–91. doi:10.1038/nature13478

8 Yilmaz M, Claiborn KC, Hotamisligil GS. De Novo Lipogenesis Products and Endogenous Lipokines. *Diabetes* 2016;**65**:1800–7. doi:10.2337/db16-0251

9 Strawford A, Antelo F, Christiansen M, *et al.* Adipose tissue triglyceride turnover, de novo lipogenesis, and cell proliferation in humans measured with {2H2O}. *Am J Physiol Endocrinol Metab* 2004;**286**:E577--588. doi:10.1152/ajpendo.00093.2003

10 Shepherd PR, Gnudi L, Tozzo E, *et al.* Adipose cell hyperplasia and enhanced glucose disposal in transgenic mice overexpressing {GLUT4} selectively in adipose tissue. *J Biol Chem* 1993;**268**:22243–6.

11 Carvalho E, Kotani K, Peroni OD, *et al.* Adipose-specific overexpression of GLUT4 reverses insulin resistance and diabetes in mice lacking GLUT4 selectively in muscle. *Am J Physiol Endocrinol Metab* 2005;**289**:E551-561. doi:10.1152/ajpendo.00116.2005

12 Nilsson E, Jansson PA, Perfilyev A, *et al.* Altered {DNA} methylation and differential expression of genes influencing metabolism and inflammation in adipose tissue from subjects with type 2 diabetes. *Diabetes* 2014;**63**:2962–76. doi:10.2337/db13-1459

13 Orozco LD, Farrell C, Hale C, *et al.* Epigenome-wide association in adipose tissue from the METSIM cohort. *Hum Mol Genet* 2018;**27**:1830–46. doi:10.1093/hmg/ddy093

For peer review only

14 Broholm C, Olsson AH, Perfilyev A, *et al.* Epigenetic programming of adipose-derived stem cells in low birthweight individuals. *Diabetologia* 2016;**59**:2664–73. doi:10.1007/s00125-016-4099-9

15 Ronn T, Volkov P, Davegardh C, *et al.* A six months exercise intervention influences the genome-wide {DNA} methylation pattern in human adipose tissue. *PLoS Genet* 2013;**9**:e1003572. doi:10.1371/journal.pgen.1003572

16 Lowe R, Slodkowicz G, Goldman N, *et al.* The human blood {DNA} methylome displays a highly distinctive profile compared with other somatic tissues. *Epigenetics* 2015;**10**:274–81. doi:10.1080/15592294.2014.1003744

17 Registrar General & Census Commissioner, India. Census of India 2011: provisional population totals-India data sheet. 2011.

18 National Family Health Survey-4. International Institute for Population Sciences. Karnataka: 2015.

19 Kumar N, Kishore J, Gupta N. Kuppuswamy's socioeconomic scale: Updating income ranges for the year 2012. *Indian J Public Health* 2012;**56**:103. doi:10.4103/0019-557X.96988

20 Matthews DR, Hosker JP, Rudenski AS, *et al.* Homeostasis model assessment: insulin resistance and beta-cell function from fasting plasma glucose and insulin concentrations in man. *Diabetologia* 1985;**28**:412–9.

21 Wallace TM, Levy JC, Matthews DR. Use and abuse of HOMA modeling. *Diabetes Care* 2004;**27**:1487–95.

22 Bharathi AV, Kurpad AV, Thomas T, *et al.* Development of food frequency questionnaires and a nutrient database for the Prospective Urban and Rural Epidemiological (PURE) pilot study in South India: methodological issues. *Asia Pac J Clin Nutr* 2008;**17**:178–85.

23 Bharathi A V, Kuriyan R, Kurpad A V, *et al.* Assessment of physical activity using accelerometry, an activity diary, the heart rate method and the Indian Migration Study questionnaire in South Indian adults. *Public Health Nutr* 2010;**13**:47. doi:10.1017/S1368980009005850

24 Harris RA, Wang T, Coarfa C, *et al.* Comparison of sequencing-based methods to profile {DNA} methylation and identification of monoallelic epigenetic modifications. *Nat Biotechnol* 2010;**28**:1097–105. doi:10.1038/nbt.1682

25 Meissner A. Reduced representation bisulfite sequencing for comparative high-resolution {DNA} methylation analysis. *Nucleic Acids Res* 2005;**33**:5868–77. doi:10.1093/nar/gki901

26 Morris AP, Voight BF, Teslovich TM, *et al.* Large-scale association analysis provides insights into the genetic architecture and pathophysiology of type 2 diabetes. *Nat Genet* 2012;**44**:981–90. doi:10.1038/ng.2383

For peer review only

27 Voight BF, Kang HM, Ding J, *et al.* The Metabochip, a Custom Genotyping Array for Genetic Studies of Metabolic, Cardiovascular, and Anthropometric Traits. *PLoS Genet* 2012;**8**:e1002793. doi:10.1371/journal.pgen.1002793

28 Ma W, Wu JHY, Wang Q, *et al.* Prospective association of fatty acids in the de novo lipogenesis pathway with risk of type 2 diabetes: the {Cardiovascular} {Health} {Study}. *Am J Clin Nutr* 2015;**101**:153–63. doi:10.3945/ajcn.114.092601

29 Mihalik SJ, Michaliszyn SF, de las Heras J, *et al.* Metabolomic Profiling of Fatty Acid and Amino Acid Metabolism in Youth With Obesity and Type 2 Diabetes: Evidence for enhanced mitochondrial oxidation. *Diabetes Care* 2012;**35**:605–11. doi:10.2337/DC11-1577

30 Yore MM, Syed I, Moraes-Vieira PM, *et al.* Discovery of a Class of Endogenous Mammalian Lipids with Anti-Diabetic and Anti-inflammatory Effects. *Cell* 2014;**159**:318–32. doi:10.1016/j.cell.2014.09.035

31 Schooneman MG, Vaz FM, Houten SM, *et al.* Acylcarnitines: Reflecting or Inflicting Insulin Resistance? *Diabetes* 2013;**62**:1–8. doi:10.2337/db12-0466

32 Roberts R, Hodson L, Dennis AL, *et al.* Markers of de novo lipogenesis in adipose tissue: associations with small adipocytes and insulin sensitivity in humans. *Diabetologia* 2009;**52**:882–90. doi:10.1007/s00125-009-1300-4

33 Lee JJ, Lambert JE, Hovhannisyan Y, *et al.* Palmitoleic acid is elevated in fatty liver disease and reflects hepatic lipogenesis. *Am J Clin Nutr* 2015;**101**:34–43. doi:10.3945/ajcn.114.092262

34 Krueger F. Trim galore. *Wrapper Tool Cutadapt FastQC Consistently Apply Qual Adapt Trimming FastQ Files* 2015;**516**:517.

35 Xi Y, Li W. BSMAP: whole genome bisulfite sequence MAPping program. *BMC Bioinformatics* 2009;**10**:232. doi:10.1186/1471-2105-10-232

36 Adusumalli S, Mohd Omar MF, Soong R, *et al.* Methodological aspects of whole-genome bisulfite sequencing analysis. *Brief Bioinform* 2015;**16**:369–79. doi:10.1093/bib/bbu016

37 Ventham NT, Kennedy NA, Adams AT, *et al.* Integrative epigenome-wide analysis demonstrates that DNA methylation may mediate genetic risk in inflammatory bowel disease. *Nat Commun* 2016;**7**:13507. doi:10.1038/ncomms13507

38 Benjamini Y, Hochberg Y. Controlling the False Discovery Rate: A Practical and Powerful Approach to Multiple Testing. *J R Stat Soc Ser B Methodol* 1995;**57**:289–300.

39 Chatterjee S, Majumder PP. Kalyani cohort – the first platform in {Eastern} {India} for longitudinal studies on health and disease parameters in peri-urban setting. *Glob Health Epidemiol Genomics* 2017;**2**:e2. doi:10.1017/gheg.2016.19

40 Nair M, Ali MK, Ajay VS, *et al.* {CARRS} {Surveillance} study: design and methods to assess burdens from multiple perspectives. *BMC Public Health* 2012;**12**:701. doi:10.1186/1471-2458-12-701

For peer review only

Figure Legends

Figure 1. The de novo lipogenesis pathway

Figure 2. The proposed link between early life determinants and lifestyle exposures on adipose DNL via epigenetic and metabolomics perturbations leading to impaired glucose tolerance.

Figure 3. The overall outline of the InDiMeT study.

Figure 4. Metabolic indicators A. percent of surveyed population with random blood glucose levels >140 mg/dl (measured in 15-54 year old men and 15-49 year old women) and B. percent of surveyed population that are overweight or obese of (i) men and (ii) women in India, Karnataka and the states with minimum and maximum values (adapted from the National Family Health Survey-4). [18]

For peer review only

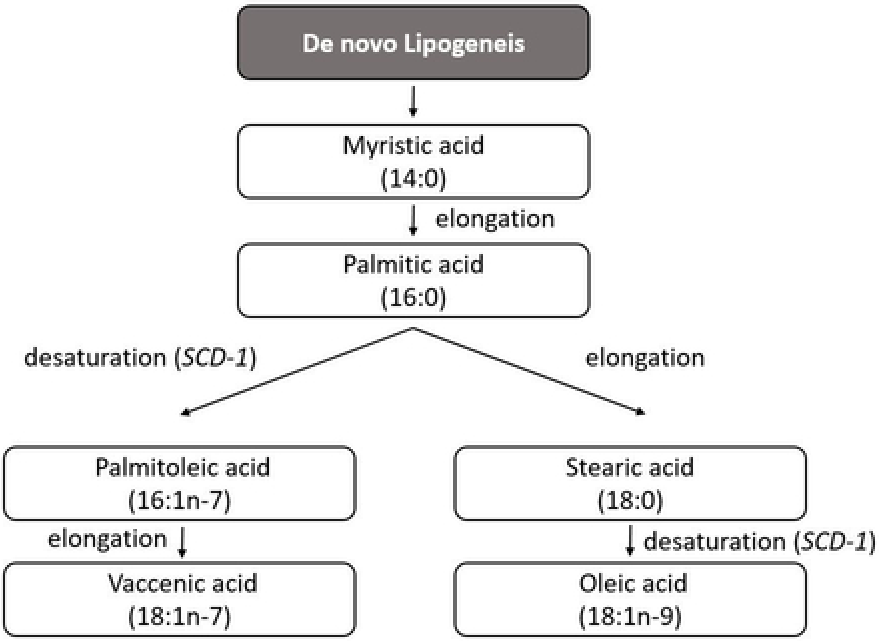

Figure 1. The de novo lipogenesis pathway

19x15mm (600 x 600 DPI)

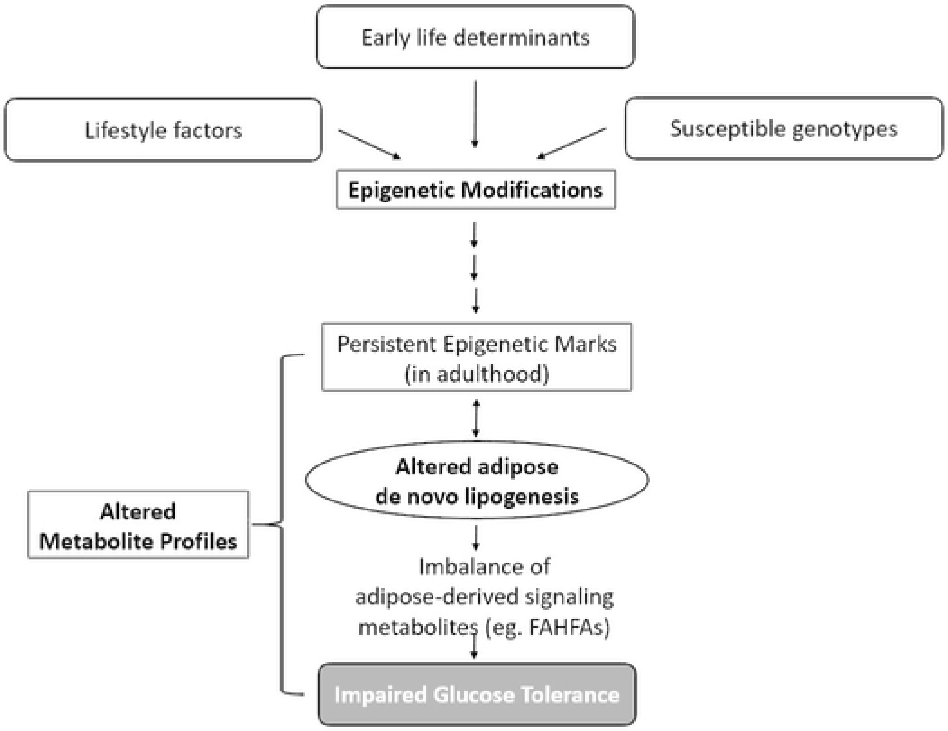

Figure 2. The proposed link between early life determinants and lifestyle exposures on adipose DNL via epigenetic and metabolomics perturbations leading to impaired glucose tolerance.

23x17mm (600 x 600 DPI)

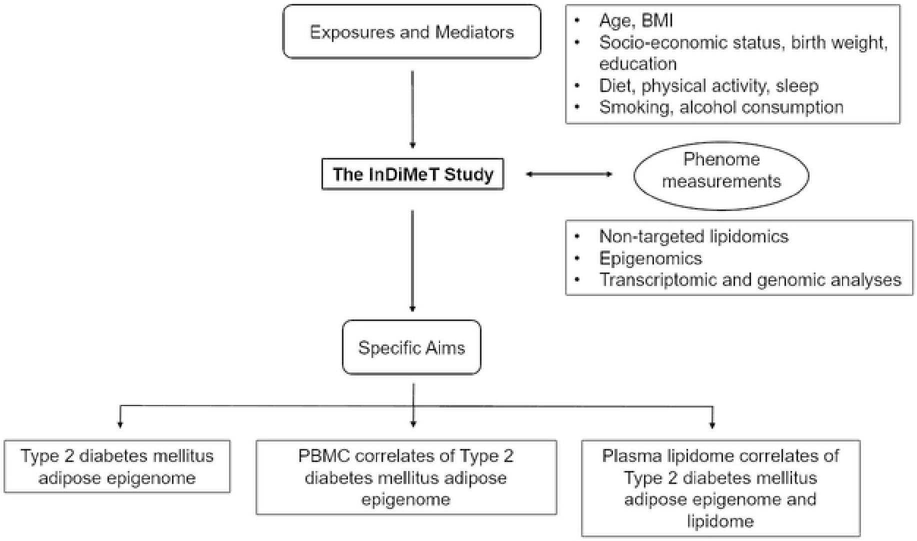

Figure 3. The overall outline of the InDiMeT study.

28x16mm (600 x 600 DPI)

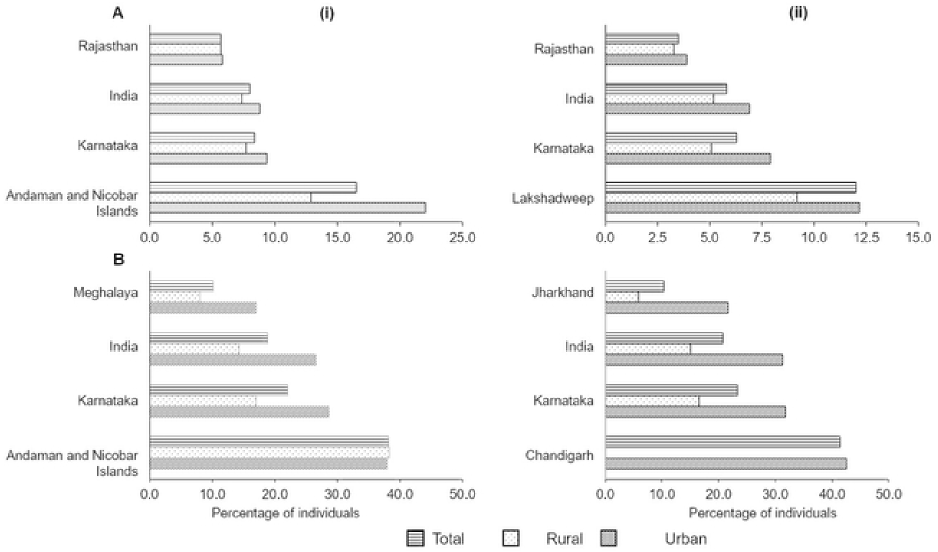

Figure 4. Metabolic indicators A. percent of surveyed population with random blood glucose levels >140 mg/dl (measured in 15-54 year old men and 15-49 year old women) and B. percent of surveyed population that are overweight or obese of (i) men and (ii) women in India, Karnataka and the states with minimum and maximum values (adapted from the National Family Health Survey-4). [18]

29x16mm (600 x 600 DPI)

BMJ Open

Protocol for a prospective, observational, deep phenotyping study on adipose epigenetic and lipidomic determinants of metabolic homeostasis in South Asian Indians: The Indian Diabetes and Metabolic Health (InDiMeT) Study

Journal:	*BMJ Open*
Manuscript ID	bmjopen-2020-043644.R1
Article Type:	Protocol
Date Submitted by the Author:	16-Jan-2021

Complete List of Authors:	Nadiger, Nikhil; St John's National Academy of Health Sciences, Division of Nutrition<br>Devi, Sarita; St John's National Academy of Health Sciences, Division of Nutrition<br>Thomas, Tinku; St John's Medical College Hospital, Division of Epidemiology and Biostatistics<br>Sivadas, Ambily; St John's National Academy of Health Sciences, Division of Nutrition<br>Raj-Kuriyan, Rebecca; St John's National Academy of Health Sciences, Division of Nutrition<br>Govindaraj, Sridar; St John's Medical College Hospital, General Surgery<br>Kurpad, Anura; St John's National Academy of Health Sciences, Division of Nutrition<br>mukhopadhyay, arpita; St John's National Academy of Health Sciences, Division of Nutrition

**Primary Subject Heading**:	Diabetes and endocrinology
Secondary Subject Heading:	Diabetes and endocrinology, Genetics and genomics
Keywords:	DIABETES & ENDOCRINOLOGY, Physiology < NATURAL SCIENCE DISCIPLINES, MOLECULAR BIOLOGY, Biochemistry < NATURAL SCIENCE DISCIPLINES, GENETICS

SCHOLARONE™
Manuscripts

I, the Submitting Author has the right to grant and does grant on behalf of all authors of the Work (as defined in the below author licence), an exclusive licence and/or a non-exclusive licence for contributions from authors who are: i) UK Crown employees; ii) where BMJ has agreed a CC-BY licence shall apply, and/or iii) in accordance with the terms applicable for US Federal Government officers or employees acting as part of their official duties; on a worldwide, perpetual, irrevocable, royalty-free basis to BMJ Publishing Group Ltd ("BMJ") its licensees and where the relevant Journal is co-owned by BMJ to the co-owners of the Journal, to publish the Work in this journal and any other BMJ products and to exploit all rights, as set out in our licence.

The Submitting Author accepts and understands that any supply made under these terms is made by BMJ to the Submitting Author unless you are acting as an employee on behalf of your employer or a postgraduate student of an affiliated institution which is paying any applicable article publishing charge ("APC") for Open Access articles. Where the Submitting Author wishes to make the Work available on an Open Access basis (and intends to pay the relevant APC), the terms of reuse of such Open Access shall be governed by a Creative Commons licence – details of these licences and which Creative Commons licence will apply to this Work are set out in our licence referred to above.

Other than as permitted in any relevant BMJ Author's Self Archiving Policies, I confirm this Work has not been accepted for publication elsewhere, is not being considered for publication elsewhere and does not duplicate material already published. I confirm all authors consent to publication of this Work and authorise the granting of this licence.

For peer review only

Protocol for a prospective, observational, deep phenotyping study on adipose epigenetic and lipidomic determinants of metabolic homeostasis in South Asian Indians: The Indian Diabetes and Metabolic Health (InDiMeT) Study

Nikhil Nadiger[1], Sarita Devi[1], Tinku Thomas[2], Ambily Sivadas[1], Rebecca Raj-Kuriyan[1], Sridar Govindaraj[3], Anura V. Kurpad[1] and Arpita Mukhopadhyay[1,*]

[1]Division of Nutrition, St. John's Research Institute, Bangalore, India

[2]Department of Biostatistics, St. John's Medical College and Hospital, Bangalore, India

[3]Department of General Surgery, St. John's Medical College and Hospital, Bangalore, India.

**Corresponding author:*

Division of Nutrition,

St. John's Research Institute,

Sarjapur Road, Bangalore, India. PIN: 560034

Phone: +91-80-49467000

Fax: +91-80-25501088

Email address: arpitam@sjri.res.in

Word count: 3734

For peer review only

ABSTRACT

Introduction

We describe the rationale and broad study design of the InDiMeT (Indian Diabetes and Metabolic Health) study, a new prospective study incorporating extensive epigenetic (DNA methylation) and lipidomic signatures to examine their association with the dysregulation of adipose de novo lipogenesis (DNL) in South Asian Indians. The InDiMeT study aims to use a case-control design to identify genetic and modifiable-environmental-lifestyle associated determinants of a) epigenomic (DNA methylome) dysregulation of adipose DNL in type 2 diabetes mellitus (T2DM) adipose tissue, b) identify correlates of epigenomic (DNA methylome) dysregulation of adipose DNL in peripheral blood mononuclear cells (PBMCs) from T2DM subjects and c) elucidate plasma lipidomic correlates of adipose DNL in T2DM that can be used as biomarkers of adipose tissue dysfunction.

Methods and analysis

The InDiMeT study will involve recruitment of 176 normoglycaemic and T2DM individuals who will be undergoing laparoscopic surgery for clinical conditions. Extensive phenotyping of the subjects will be conducted and DNA methylome and lipidomic measurements will be made. The adipose DNL pathway genes are likely to be hypermethylated in T2DM patients with corresponding reduction of gene expression. Correlates of epigenomic (DNA methylome) dysregulation of adipose DNL pathway in PBMCs and their adipose and plasma lipidomic signatures in T2DM subjects could act as early markers of development of T2DM.

Ethics and Dissemination

For the InDiMeT study, ethical approval for addressing the specific aims has been obtained from the Institutional Ethics Committee, St. John's Medical College and Hospital, St. John's National Academy of Health Sciences, Bangalore. Findings from this study will be disseminated through scientific publications in peer-reviewed journals, research conferences

For peer review only

For peer review only - http://bmjopen.bmj.com/site/about/guidelines.xhtml

and via presentations to stakeholders, patients, clinicians, public and policymakers through appropriate channels.

Keywords: type 2 diabetes mellitus, South Asian Indians, genotyping, epigenetics, DNA methylome, lipidome, pathophysiology, adipose tissue, de novo lipogenesis

For peer review only

For peer review only - http://bmjopen.bmj.com/site/about/guidelines.xhtml

ARTICLE SUMMARY

Strengths and limitations of this study

- The InDiMeT study will assess the epigenome (DNA methylome)-lipidome connection in relation to their contribution to adipose de novo lipogenesis (DNL) and metabolic homeostasis.
- The prospective case-control study design will permit us to assess the blood epigenetic (DNA methylation) and lipidomic markers that exhibit correlations with adipose DNL dysregulation and can also potentially serve as minimally invasive early biomarkers of dysglycemia.
- Understanding the epigenetic (DNA methylation) mechanisms and their associated lipidomic profiles related to dysregulated adipose DNL in South Asian Indians will provide the rationale for effective interventional strategies targeted to this population and discriminative diagnostic/prognostic biomarkers for prevention of T2DM in this high-risk population.
- The small sample size of 176 individuals is a likely limitation of the study.
- The subjects being recruited are the individuals who come for treatment of their preexisting clinical conditions and could not be a random representation of the total population.

INTRODUCTION

India is experiencing a type 2 diabetes mellitus (T2DM) epidemic, attributed to rapid sociodemographic and nutritional changes.[1] Younger, non-obese South Asian Indians develop insulin resistance, attributed to inappropriately elevated fat accretion.[2] An important reason for elevated/ectopic fat deposition in South Asian Indians may be their uniquely high carbohydrate diets (~65 energy%), which has been associated with elevated risk of T2DM,

For peer review only - http://bmjopen.bmj.com/site/about/guidelines.xhtml

likely due to induction of hepatic insulin resistance through increased hepatic de novo lipogenesis (DNL, **Figure 1**).[3–5]

Unlike hepatic DNL, adipose DNL is understood to be associated with preservation of insulin sensitivity and an overall favourable metabolic phenotype ('metabotype').[6] Strawford et al reported negative correlations between adipose DNL and plasma glucose in humans.[7] Mice overexpressing adipose-specific GLUT4 are obese but paradoxically insulin sensitive, likely mediated by elevated adipocyte glucose influx-induced DNL.[8] Further, adipose-specific GLUT4 overexpression can rescue muscle-specific GLUT4 knockout-mediated insulin resistance, in a background of increased adiposity.[9] Therefore, it can be posited that white adipose tissue (AT) versus hepatic DNL may have opposed effects with respect to obesity-associated metabolic complications. Thus, understanding the contribution of adipose DNL in healthy versus T2DM-associated obesity is critical.

Understanding the epigenetic (DNA methylation) mechanisms and their associated lipidome profiles related to exacerbated adipose DNL in South Asian Indians will further provide the rationale for effective interventional strategies targeted to this population and discriminative diagnostic/prognostic biomarkers for prevention of T2DM in this high-risk population.

Rationale and Specific Aims

The overarching goal of the Indian Diabetes and Metabolic Health (InDiMeT) study is to identify etiologically valuable determinants that affect susceptibility to type 2 diabetes at individual, sub-group and population level by testing the hypothesis that the epigenetic (DNA methylation) dysregulation of adipose DNL induced by high carbohydrate diet and environmental factors is an important step in the development adipose tissue dysfunction leading to type 2 diabetes (**Figure 2**) in India. The InDiMeT study proposes to achieve this by

For peer review only - http://bmjopen.bmj.com/site/about/guidelines.xhtml

conducting detailed phenotyping and phenome measurements in a prospective manner in a high-risk population setting. Though animal studies have underlined the epigenetic changes in the adipose associated, few have investigated adipose epigenetics in humans and none in the context of adipose DNL dysregulation and T2DM [10–12] which will help to identify epigenomic dysregulation of adipose DNL in T2DM AT. Although DNA methylation signatures are specific to tissue and cell types, only ~38% of the DNA methylation probes have been reported to be differentially methylated in blood in a tissue-specific fashion, implying likelihood of the rest ~62% probes being conservatively methylated between blood and other tissues.[13] Since blood is an easily available tissue, correlates of epigenomic (DNA methylome) dysregulation of adipose DNL in peripheral blood mononuclear cells (PBMCs) from T2DM subjects will be evaluated to validate the potential use of PBMCs as surrogates of AT dysfunction.

The overall outline of the InDiMeT study is depicted in **Figure 3**. The specific aims of this study are:

1. To identify epigenomic (DNA methylome) dysregulation of adipose de novo lipogenesis (DNL) in T2DM adipose tissue
2. To identify correlates of epigenomic (DNA methylome) dysregulation of adipose DNL in PBMCs from T2DM subjects
3. To elucidate plasma lipidomic correlates of adipose DNL in T2DM that can be used as biomarkers of adipose tissue dysfunction

METHODS AND ANALYSIS

Study setting

The InDiMeT study is being conducted at St. John's Medical College and Hospital, a tertiary care hospital in Bangalore district, India. It is surrounded by the state of Tamil Nadu in

For peer review only - http://bmjopen.bmj.com/site/about/guidelines.xhtml

the southeast, the Bangalore Rural district in the northeast and northwest, and the Ramanagara district in the southwest.[14] As per the Indian 2011 Census, the Bangalore district recorded a decadal growth rate increase of 12% in the decade of 2001-2011 compared to the previous decade. This growth was ~4 times higher in the urban areas compared to the rural ones. The sex ratio of the Bangalore district was 916, an 8-point increase compared to the 2001 Census. The literacy rate was 88% while the work participation rate was 44%, higher for males at 62% compared to females at 25%. As per the National Family Health Survey-4, one-third of the women and 26% of the men in Bangalore (Bangalore urban district) are overweight or obese.[15] In Bangalore urban district, prevalence of elevated random blood sugar measurements stands at 8.3% amongst women and 10.9% amongst men which compares to Karnataka state-wide figures of 6.3% for women and 8.4% for men respectively (**Figure 4**).

Study design and population

The InDiMeT study has been designed as a prospective tertiary care hospital-based cohort study. 176 normoglycemic and T2DM individuals will be recruited into the study after explanation of details of the study and obtaining informed consent at the department of General surgery, St. John's Medical College and Hospital, St. John's National Academy of Health Sciences, Bangalore. Dissemination of information about the broader study goals and likely benefits to the community as well as engagement of potential subjects from the hospital was initiated in August 2020. Recruitment of subjects commenced from November 19th 2020, after ethical approval for the study had been obtained from the Institutional Ethics Committee. Recruitment of subjects will continue till the recruitment target (176 individuals) is achieved. Strategies elaborated below are being employed for recruitment:

- Chart reviews – From the existing group of patients coming in to the Department of General Surgery, St. John's Medical College and Hospital, subjects are screened and then contacted to request for their participation.

For peer review only - http://bmjopen.bmj.com/site/about/guidelines.xhtml

- Databases – From the database of patients coming to the St. John's Medical College and Hospital, those who are eligible to participate in the study are contacted for possible recruitment.

Inclusion criteria: 35-60 years age; normoglycaemic and T2DM; normal and high BMI; men and women who are undergoing laparoscopic surgeries for their clinical conditions such as cholecystectomies, hernia or splenectomy are invited to participate in the study.

Exclusion criteria: Age outside the range of 35-60 years, individuals who are unwilling to participate in the study, individuals participating in any other study and those who tested positive for hepatitis (HBsAg), HIV or syphilis (VDRL) infections. Those who have serious pre-existing medical condition, those undergoing laparoscopic surgery for clinical conditions other than cholecystectomies, hernia or splenectomy will be excluded, and these will be defined as conditions that require chronic or daily medical therapy, including medications, for T2DM, connective tissue diseases, inflammatory bowel disease, active tuberculosis and symptomatic heart disease.

Sample size calculation: Based on the study by Nilsson et al, the sample size required to observe a mean difference of 7% in DNA methylation in AT with an SD of 10% between T2DM and normoglycaemic controls with 90% power and 5% level of significance is 44 per group. As AT DNA methylation differences could be associated with BMI of the subjects, 44 individuals will be recruited in the normal (BMI<25 kg/m^2) and high BMI (BMI≥25 kg/m^2) groups of normoglycaemic controls and T2DM (35-60 years, men and women).[10]

Data collection

Socio-demographic data collection

For peer review only - http://bmjopen.bmj.com/site/about/guidelines.xhtml

Detailed socio-demographic data will be collected by trained personnel using well-structured questionnaires as elaborated in **Table 1**. The questionnaire will be provided and explained in English or local language in which the subject is most comfortable.

Socioeconomic status will be calculated based on scoring relevant for the Indian scenario.[16]

Table 1. Exposure and outcome measurements within the InDiMeT Study.

**General questionnaires**
Socio-demographic
Age, ethnicity and religion, family composition, education, employment status and type, household income, socioeconomic status
Medical history
Medical history related to birth weight, chronic and infectious diseases, last one month's morbidity conditions, current and past usage of tobacco (cigarettes, beedis or chewing tobacco) and alcohol, family history of diabetes and cardiovascular disease, details on most recent routine clinical investigations (haemoglobin, fasting/random/post-prandial glucose, OGTT glucose, HbA1C, serum insulin and C-peptide, urea, creatinine, Calcium, Phosphorus, TSH, Uric acid, serum cholesterol, HDL, LDL, VLDL, triglycerides, serum amylase and lipase, total protein, albumin, total bilirubin, direct and indirect bilirubin, SGOT, SGPT, alkaline phosphatase, GGT, sodium, potassium, chloride, routine urine test, urine albumin)
Medication and supplement use
Type, brand, dose and duration of medication, vitamin and mineral supplements (clinician prescribed or over the counter)
Dietary habits
3 months food-frequency questionnaire

For peer review only

For peer review only - http://bmjopen.bmj.com/site/about/guidelines.xhtml

Physical activity	
	Daily, weekly and monthly frequency of sports activities, manual labour related hobbies and household chores, sedentary activities; occupational classification in terms of physical activity; sleep habits
**Anthropometry, body composition measurements and laboratory assessments**	
Vital sign measurements	
	Blood pressure, heart rate and pulse
Body composition and anthropometry measurements	
	DXA scanning, measurement of height and weight
Laboratory assessments	
	Blood investigations: Blood glucose level, Glycosylated Haemoglobin (HbA1C), serum insulin, C-peptide, C Reactive Protein (CRP), Glucagon-like peptide (GLP) and Glucose-dependent insulinotropic polypeptide (GIP)
**Biobanking**	
Collection of blood samples	
	Separation and storage of plasma for lipidomic analyses
Collection of abdominal visceral adipose tissue	
	Done during laparoscopic surgeries; collection and storage of adipose tissue for lipidomic, epigenomic, genomic and gene expression analyses
**Phenome measurements**	
Non-targeted lipidomic analyses	
	on adipose tissue and plasma samples
Epigenomic analysis	
	on adipose tissue samples and PBMCs
Gene expression analysis	

For peer review only - http://bmjopen.bmj.com/site/about/guidelines.xhtml

on adipose tissue samples

Genomic analysis

on PBMCs

OGTT: oral glucose tolerance test, TSH: thyroid-stimulating hormone, HDL: high-density lipoprotein, LDL: low-density lipoprotein, VLDL: very low-density lipoprotein, SGOT: serum glutamic oxaloacetic transaminase, SGPT: serum glutamic pyruvic transaminase, GGT: gamma-glutamyl transferase, DXA: dual-energy X-ray absorptiometry, GAD: glutamic acid decarboxylase, PBMC: peripheral blood mononuclear cells

Medical history and medication and supplement use data collection

Participants will be asked regarding their birth weight, recent morbidity conditions and the medications and supplements they are currently taking. Details of the type and usage of these medications and supplements will be recorded by trained staff (Table 1). Participants will be requested to provide these details from the prescriptions or their hospital records will be sought after.

Vital sign measurements

Participants will be requested to relax and then blood pressure, heart rate and pulse will be measured and recorded by trained staff.

Body composition measurement by DXA scanning and anthropometry

Participants will be weighed in minimal clothing using a digital scale, to a precision of 0.1 kg. The height of the subjects will be recorded to the nearest 0.1 cm. Whole body and regional body composition will also be estimated using DXA (DPXMD 7254, Lunar

For peer review only

For peer review only - http://bmjopen.bmj.com/site/about/guidelines.xhtml

Corporation, Madison, WI). The total body fat (BF) will be measured and both fat and muscle (ALST) will be expressed as a percentage of body weight (% BF and % ALST).

Laboratory assessments

Blood samples before and after their surgery will be collected from the participants. The blood sample collected before surgery will be a fasting sample as the participants abstain from eating anything 10 hours before surgery, as a pre-operative requirement for surgery. These samples will be utilized for assessments as elaborated in **Table 1**. Muscle insulin sensitivity and β-cell function will be assessed by the homeostasis model assessment-of insulin resistance (HOMA-IR) and of β-cell function (HOMA-B) respectively.[17,18]

Lifestyle factors

Dietary data collection

The dietary intake will be assessed using a validated food frequency questionnaire (FFQ). The FFQ will be administered by trained personnel to obtain information about the habitual dietary intake for the preceding 3 months.[19] The nutrients and food groups will be estimated for all the foods listed in the FFQ and summed to obtain the total nutrient or food group intake per day for an individual. Nutrient information will be assessed for 27 macronutrients and micronutrients.

Physical activity data collection

The habitual physical activity and sleep habits of the subjects will be assessed by detailed and validated physical activity questionnaire.[20]

Biobanking

For peer review only

Blood collection and storage

Blood samples will be collected before and after the surgery by phlebotomy in EDTA/serum tubes, kept on ice, and immediately processed for aliquoting and storage at -80°C.

Abdominal visceral adipose tissue collection and storage

Intraabdominal omental adipose tissue will be collected during laparoscopic surgery with clinical indications of cholecystectomy, meshplasty (hernia) or splenectomy. The collected tissue will be appropriately stored for phenome measurements (epigenomic and non-targeted lipidomic analyses) as described below.

Phenome measurements

Epigenomic (DNA methylome) analysis

Nucleic acids will be extracted from peripheral blood mononuclear cells (PBMCs) and adipose tissue. Epigenome-wide assessment of DNA methylation levels in CpG islands will be done by reduced representation bisulfite sequencing (RRBS) after bisulfite conversion.[10,21,22]

Gene expression by quantitative real-time PCR (qPCR) will be performed with SYBR Green chemistry for the top ten differentially methylated loci and for the adipose DNL pathway related genes: *MLXIPL, SREBP1, ACLY, ACC1, FASN, CPT1A, CPT1B, SCD* and *ELOVL6*. While lysis of citrate to acetyl CoA, the initial substrate of lipogenesis, is catalyzed by ATP-citrate lyase (ACLY) in the cytosol, cytosolic acetyl-CoA carboxylase (ACC1) condenses acetyl CoA into malonyl CoA.[23] Fatty acid synthase (FASN) then sequentially condenses 2-carbon units from malonyl CoA to form myristic acid and palmitic acid. Palmitic acid can then either undergo further elongation by elongase 6 (ELOVL6) or desaturation by stearoyl-CoA

For peer review only

For peer review only - http://bmjopen.bmj.com/site/about/guidelines.xhtml

desaturate (SCD). Carnitine palmitoyltransferase 1A and 1B (CPT1A and CPT1B), SREBP1 and MLXIPL are involved in regulation of the DNL pathway. CPT1A and CPT1B are required for transport of long-chain fatty acyl-CoAs from cytosol to mitochondria for the beta-oxidation pathway. MLX Interacting Protein Like (MLXIPL), also known as Carbohydrate-Responsive Element-Binding Protein (CHREBP) primarily and Sterol Regulatory Element Binding Transcription Factor 1 (SREBP1), to a lesser extent, control and activate transcription of multiple DNL genes such as ACC1 and ELOVL6 in adipocytes.[24]

Genotyping of the subjects will be done using Metabochip (a custom genotyping array for metabolic and cardiovascular traits) or equivalent single nucleotide polymorphism (SNP) array, such as the Infinium Global Screening Array (Illumina Inc., USA).[25,26]

Non-targeted lipidomic analysis

Plasma and omental AT samples will be used for non-targeted lipidomic analysis. Samples will be spiked with SPLASH™ Lipidomix Mass Spec standard (Avanti Polar Lipids, USA) before lipid extraction and reconstitution. Non-targeted lipidomic analysis will be performed on a high-resolution analytical platform consisting a Vanquish Flex Binary UHPLC coupled to a Q Exactive Orbitrap, high-resolution accurate-mass spectrometer (LC-HRAM-MS, Thermo Scientific, USA). High resolution accurate mass data generated by Thermo Scientific Q Exactive Orbitrap will be identified and quantified by Thermo Scientific Lipid Search™ software. The following lipid species are of special interest: palmitic acid, stearic acid, oleic acid, myristic acid, palmitoleic acid, acyl carnitines, palmitic acid esters of hydroxyl stearic acids (PAHSAs), furan fatty acid metabolite 3-carboxy-4-methyl-5-propyl-2-furanpropanoic acid (CMPF).[27–30]

Assessment of adipose DNL will be done by determining AT triglyceride (TG) content of the fatty acids myristic acid (14:0), palmitic acid (16:0) and stearic acid (18:0) that have

For peer review only - http://bmjopen.bmj.com/site/about/guidelines.xhtml

been reported to be correlated with plasma non-esterified fatty acids (NEFA) 14:0 and 18:0 but not with plasma NEFA 15:0, that is exclusively derived from dietary dairy sources.[31] Further, AT lipid species (from non-targeted lipidomic data) that positively correlate with transcript abundances of adipose DNL pathway genes will be identified as indicators of adipose DNL. It should be noted here that though adipose tissue myristic acid, palmitic acid and steric acid contents are not direct measures of adipose DNL, they represent end products of DNL and have been reported to be correlated to expression of genes involved in adipose DNL.[31]

Plasma NEFA correlates of adipose DNL will be identified in the T2DM and normoglycaemic subjects by correlation analysis of plasma lipidomic data with adipose DNL measures [AT triglyceride (TG) content of the fatty acids myristic acid (14:0), palmitic acid (16:0) and stearic acid (18:0)]. This is a novel strategy for estimating adipose DNL and identifying plasma correlates of adipose DNL as such indirect methods do not exist at present and the direct stable isotope based method of measuring DNL primarily measures post-prandial hepatic DNL rates.[32]

Data analysis plan

Continuous data will be presented as either mean ± SD or median (quartile1, quartile 3). The normality of distribution will be examined using the Shapiro-Wilk test and Q-Q plots. Demographic and clinical characteristics will be compared between cases of T2DM and controls by Student's t-test, if normally distributed or Mann-Whitney U test, if non-normally distributed, for continuous variables and by Chi-square test for categorical variables.

For analysis of RRBS data, the read sequences from the RRBS libraries will be adapted/quality trimmed using Trim Galore[33] and aligned to the reference human genome using a wildcard mapper like BSMap.[34] DNA methylation scores (β values) will be calculated from the aligned reads.[35] Differentially methylated position (DMP) analysis will

For peer review only - http://bmjopen.bmj.com/site/about/guidelines.xhtml

282 be conducted using limma software while adjusting for cell proportions (for PBMCs) and
283 including age and sex as covariates.[36] Statistical significance will be set at P<0.05 following
284 adjustment for multiple testing using the Benjamini Hochberg false discovery rate (FDR).[37]
285 Differentially methylated regions (DMRs) will be defined as three or more contiguous probes
286 within a 2 kb distance, each sharing the same direction of methylation change, each achieving
287 a Benjamini Hochberg FDR corrected P<0.05 in DMP analysis. Enrichment analysis will be
288 performed to understand the top significantly deregulated gene ontologies and pathways.
289 Additionally, in-depth analysis of methylation status of the CpG islands in the promoter and
290 coding regions of genes associated with the DNL pathway will be conducted.

291 Association studies between the CpG methylated regions and clinical traits will be
292 performed using linear mixed-model package pyLMM and significant associations will be
293 determined using Bonferroni corrected p-value cut-offs.

294 Gene expression values will be estimated by normalizing target gene expression against
295 expression of housekeeping genes followed by comparison of target gene expression between
296 the groups.

297 Methylation Quantitative Trait Loci (mQTLs) will be identified by testing for
298 association between methylation levels and genotypes of the samples using FDR correction.

299 Correlation-based clustering analysis of the lipid profiles will be performed and
300 visualized as heatmaps to evaluate the interrelationships between the lipid species and for
301 identifying signatures that differentiate between T2DM and control samples using supervised
302 and unsupervised approaches. For heatmap analysis, only significantly altered features
303 (significant alterations will be defined) will be analyzed and data will be organized using a
304 Euclidean distance map and complete clustering algorithm. Random forest will be used to
305 assess the classification performance of the lipidomics signatures. The significantly perturbed
306 pathways will be identified based on the differentially regulated lipids. For random forest and

For peer review only - http://bmjopen.bmj.com/site/about/guidelines.xhtml

pathway impact analyses, data will be filtered using the interquartile range (IQR) function and scaled using auto-scaling, in which values are mean-centered and divided by the standard deviation of each variable. For the random forest analysis, the number of predictors to try for each node will be set to the square root of the total number of variables.

To identify the adipose lipid species that correlate with transcript abundances of genes in the adipose DNL pathway, first correlation analysis (Pearson's correlation, for normally distributed data and Spearman's correlation, for non-normally distributed data) will be conducted. This will be followed by regression analysis, with levels of lipid species exhibiting significant correlations as dependent variable and transcript abundance of individual genes in the adipose DNL pathway as independent variables, adjusting for probable confounders such as BMI and sex, if found to be significantly correlated with the dependent variables.

All statistical analysis will be processed and analyzed using a combination of SPSS Version 25 (IBM, USA), STATA 14 (StataCorp, USA) and R (R Foundation for Statistical Computing, Vienna).

Patient and public involvement

Patients and general public were not invited to comment on the study design and were not consulted to formulate study outcomes. Results for the InDiMeT study will be communicated to the patients and public in two complementary ways. Data related to individual participants of the study will be shared with them, if deemed beneficial for improving their health status, in consultation with clinician collaborators on this study and specific participant or their primary care providers. This is mandated by our Institutional Ethics Committee as well. The overall study results will be disseminated to participating individuals and public via appropriate forums for public science outreach.

For peer review only - http://bmjopen.bmj.com/site/about/guidelines.xhtml

ETHICS AND DISSEMINATION

Ethical considerations and informed consent

Institutional ethical approval for the study has been obtained from the Institutional Ethics Committee (IEC), St. John's Medical College and Hospital, St. John's National Academy of Health Sciences, Bangalore (IEC Study Ref No. 246/2020, IEC Study title: "De novo lipogenesis in adiposity and glycaemic control: Epigenetic mechanisms and associated metabolite profiles"). Subject recruitment has been initiated from November 19th 2020.

The objectives and aims of the study are being explained in subject's preferred local language to interested volunteers who fulfill the inclusion criteria. The Patient Information Sheet contains relevant and specific information about the study, collection and storage of samples, genetic analysis and safe storage of subject data. A written, informed consent is being obtained from the subjects before the start of the protocol.

Data storage, management and protection

Data and resources generated as part of this study will be managed, stored and shared in line with Institutional, State and National policies. In brief, specifics are as follows:

- *Organization*: Conventions for naming and storage organization of data files as well as samples from human subjects (adipose and blood) will be set up, documented and circulated amongst study team members at the beginning of the study.
- *Storage*: Data will be stored in a secure manner in three copies in two different media. Back-up copies of data will be accompanied with metadata that describe the stored data in a standard, understandable format. Data storage and protection support will be obtained from the Institutional Information Technology team.
- *Physical samples*: Samples from the study subjects will be processed and stored as per the experimental protocols. Samples in excess of the requirements of the study

For peer review only - http://bmjopen.bmj.com/site/about/guidelines.xhtml

objectives will be stored appropriately as per the Institutional policies as well as policies of the peer-reviewed journals where findings from the study will be submitted for possible publications.

Dissemination of data

Sequence data generated from this study will be deposited in GenBank and epigenetic data in Gene Expression Omnibus, while raw and processed lipidomic data will be deposited in appropriate repositories such as Metabolomics Workbench or MetaboLights during the time of publication. Findings from the InDiMeT study will be appropriately communicated in refereed journals and forums for scientific and community science outreach.

SIGNIFICANCE AND OUTLOOK

The InDiMeT study will provide the base and act as a robust tool for mechanistic interpretations of public health and epidemiological aspects of developing T2DM and its complications in the Indian setting. Though a few large, prospective cohort studies to identify the determinants of T2DM are being conducted in India, to the best of our knowledge, none of them involve stated deep phenotyping or extensive phenome measurements of the participants.[38,39] This study was conceptualized to fill this niche in this country. The carefully monitored and quality-controlled collection and archiving of biological samples, along with availability of anthropometric, socio-demographic, nutritional and medical data on the participants will aid toward planning and implementation of an array of hypothesis-driven mechanistic studies.

The InDiMeT study will not be without limitations. Notable ones will include the low probability of participation by individuals of specific subsets of the population due to the voluntary nature of participation. The subjects being recruited are the individuals who come

For peer review only - http://bmjopen.bmj.com/site/about/guidelines.xhtml

for treatment of their preexisting clinical conditions could not be a random representation of the total population. Further, the study setting being a tertiary care hospital in a large, urban area in India, a country known for its large regional variations in cuisine and culture-associated dietary habits, composition and amounts of habitual dietary intakes of the study subjects are likely to be heterogenous. These factors could act as confounders and limit our ability to detect differences in DNA methylation and lipid profiles that are of low effect sizes. Detailed recording of these factors as proposed, will however help us in understanding if these factors do act as confounders in our study and allow us and others to improve design of similar studies in future.

Acknowledgements

The contribution of research fellow Ms. Bajanai Nongkhlaw, research assistant Ms. Beena Bose and nutritionist Mr. J. Jayakumar is gratefully acknowledged.

Authors' contributions

The authors of the manuscript and their individual contributions towards the manuscript are as follows. AM, TT and AVK: designed the research; NN, SD and SG: conduct of the study; AM, TT, AS, RR-K and AVK: analysis of the data, performing statistical analyses and interpretation of the results; AM and AVK: obtained funding; AM: had the idea for and led writing of the manuscript and had primary responsibility for the final content of the manuscript; and all authors critically reviewed the manuscript, read and approved the final version of the manuscript.

Funding

For peer review only - http://bmjopen.bmj.com/site/about/guidelines.xhtml

This study is supported by the Wellcome Trust/DBT India Alliance Fellowship awarded to AM [grant number IA/CPHI/19/1/504593] and to AVK [grant number IA/M/14/1/501681] and by the Department of Biotechnology, Government of India [Grant Sanction order BT/PR24342/PFN/20/1314/2017 to AM and AVK].

Competing interests

None declared.

For peer review only

References

1 World Health Organization. Global Health Estimates: Deaths by Cause, Age, Sex and Country, 2000-2012. Geneva: 2014.

2 Misra A, Madhavan M, Vikram NK, *et al.* Simple anthropometric measures identify fasting hyperinsulinemia and clustering of cardiovascular risk factors in Asian Indian adolescents. *Metabolism* 2006;**55**:1569–73. doi:10.1016/j.metabol.2006.06.029

3 Mohan V, Radhika G, Sathya RM, *et al.* Dietary carbohydrates, glycaemic load, food groups and newly detected type 2 diabetes among urban Asian Indian population in Chennai, India (Chennai Urban Rural Epidemiology Study 59). *Br J Nutr* 2009;**102**:1498–506. doi:10.1017/S0007114509990468

4 Acheson KJ, Schutz Y, Bessard T, *et al.* Carbohydrate metabolism and de novo lipogenesis in human obesity. *Am J Clin Nutr* 1987;**45**:78–85.

5 Perry RJ, Samuel VT, Petersen KF, *et al.* The role of hepatic lipids in hepatic insulin resistance and type 2 diabetes. *Nature* 2014;**510**:84–91. doi:10.1038/nature13478

6 Yilmaz M, Claiborn KC, Hotamisligil GS. De Novo Lipogenesis Products and Endogenous Lipokines. *Diabetes* 2016;**65**:1800–7. doi:10.2337/db16-0251

7 Strawford A, Antelo F, Christiansen M, *et al.* Adipose tissue triglyceride turnover, de novo lipogenesis, and cell proliferation in humans measured with 2 H 2 O. *Am J Physiol Metab* 2004;**286**:E577–88. doi:10.1152/ajpendo.00093.2003

8 Shepherd PR, Gnudi L, Tozzo E, *et al.* Adipose cell hyperplasia and enhanced glucose disposal in transgenic mice overexpressing GLUT4 selectively in adipose tissue. *J Biol Chem* 1993;**268**:22243–6. doi:10.1007/s00125-008-1025-9

9 Carvalho E, Kotani K, Peroni OD, *et al.* Adipose-specific overexpression of GLUT4 reverses insulin resistance and diabetes in mice lacking GLUT4 selectively in muscle. *Am J Physiol Endocrinol Metab* 2005;**289**:E551–61. doi:10.1152/ajpendo.00116.2005

For peer review only

For peer review only - http://bmjopen.bmj.com/site/about/guidelines.xhtml

10 Nilsson E, Jansson PA, Perfilyev A, *et al.* Altered DNA Methylation and Differential Expression of Genes Influencing Metabolism and Inflammation in Adipose Tissue From Subjects With Type 2 Diabetes. *Diabetes* 2014;**63**:2962–76. doi:10.2337/db13-1459

11 Orozco LD, Farrell C, Hale C, *et al.* Epigenome-wide association in adipose tissue from the METSIM cohort. *Hum Mol Genet* 2018;**27**:1830–46. doi:10.1093/hmg/ddy093

12 Broholm C, Olsson AH, Perfilyev A, *et al.* Epigenetic programming of adipose-derived stem cells in low birthweight individuals. *Diabetologia* 2016;**59**:2664–73. doi:10.1007/s00125-016-4099-9

13 Lowe R, Slodkowicz G, Goldman N, *et al.* The human blood DNA methylome displays a highly distinctive profile compared with other somatic tissues. *Epigenetics* 2015;**10**:274–81. doi:10.1080/15592294.2014.1003744

14 Register General I. Census of India 2011: provisional population totals-India data sheet. 2011.

15 Indian Institute for Population Sciences. National Family Health Survey - 4. Mumbai: 2017. http://rchiips.org/NFHS/factsheet_NFHS-4.shtml (accessed 12 Aug 2019).

16 Kumar N, Kishore J, Gupta N. Kuppuswamy's socioeconomic scale: Updating income ranges for the year 2012. *Indian J Public Heal* 2012;**56**:103. doi:10.4103/0019-557X.96988

17 Matthews DR, Hosker JP, Rudenski AS, *et al.* Homeostasis model assessment: insulin resistance and beta-cell function from fasting plasma glucose and insulin concentrations in man. *Diabetologia* 1985;**28**:412–9.http://www.ncbi.nlm.nih.gov/pubmed/3899825

18 Wallace TM, Levy JC, Matthews DR. Use and abuse of HOMA modeling. *Diabetes*

For peer review only

For peer review only - http://bmjopen.bmj.com/site/about/guidelines.xhtml

Care 2004;**27**:1487–95.http://www.ncbi.nlm.nih.gov/pubmed/15161807

19 Bharathi AV, Kurpad AV, Thomas T, *et al.* Development of food frequency questionnaires and a nutrient database for the Prospective Urban and Rural Epidemiological (PURE) pilot study in South India: methodological issues. *Asia Pac J Clin Nutr* 2008;**17**:178–85.

20 Bharathi A V, Kuriyan R, Kurpad A V, *et al.* Assessment of physical activity using accelerometry, an activity diary, the heart rate method and the Indian Migration Study questionnaire in South Indian adults. *Public Heal Nutr* 2010;**13**:47. doi:10.1017/S1368980009005850

21 Harris RA, Wang T, Coarfa C, *et al.* Comparison of sequencing-based methods to profile DNA methylation and identification of monoallelic epigenetic modifications. *Nat Biotech* 2010;**28**:1097–105. doi:10.1038/nbt.1682

22 Meissner A. Reduced representation bisulfite sequencing for comparative high-resolution DNA methylation analysis. *Nucleic Acids Res* 2005;**33**:5868–77. doi:10.1093/nar/gki901

23 Strable MS, Ntambi JM. Genetic control of de novo lipogenesis: role in diet-induced obesity. *Crit Rev Biochem Mol Biol* 2010;**45**:199–214. doi:10.3109/10409231003667500

24 Song Z, Xiaoli A, Yang F. Regulation and Metabolic Significance of De Novo Lipogenesis in Adipose Tissues. *Nutrients* 2018;**10**:1383. doi:10.3390/nu10101383

25 Morris AP, Voight BF, Teslovich TM, *et al.* Large-scale association analysis provides insights into the genetic architecture and pathophysiology of type 2 diabetes. *Nat Genet* 2012;**44**:981–90. doi:10.1038/ng.2383

26 Voight BF, Kang HM, Ding J, *et al.* The Metabochip, a Custom Genotyping Array for Genetic Studies of Metabolic, Cardiovascular, and Anthropometric Traits. *PLoS Genet*

For peer review only - http://bmjopen.bmj.com/site/about/guidelines.xhtml

2012;**8**:e1002793. doi:10.1371/journal.pgen.1002793

27 Wenjie M, Wu JHY, Wang Q, *et al.* Prospective association of fatty acids in the de novo lipogenesis pathway with risk of type 2 diabetes: The Cardiovascular Health Study. *Am J Clin Nutr* 2015;**101**:153–63. doi:10.3945/ajcn.114.092601

28 Mihalik SJ, Michaliszyn SF, De Las Heras J, *et al.* Metabolomic profiling of fatty acid and amino acid metabolism in youth with obesity and type 2 diabetes: Evidence for enhanced mitochondrial oxidation. *Diabetes Care* 2012;**35**:605–11. doi:10.2337/DC11-1577

29 Yore MM, Syed I, Moraes-Vieira PM, *et al.* Discovery of a Class of Endogenous Mammalian Lipids with Anti-Diabetic and Anti-inflammatory Effects. *Cell* 2014;**159**:318–32. doi:10.1016/j.cell.2014.09.035

30 Schooneman MG, Vaz FM, Houten SM, *et al.* Acylcarnitines: Reflecting or Inflicting Insulin Resistance? *Diabetes* 2013;**62**:1–8. doi:10.2337/db12-0466

31 Roberts R, Hodson L, Dennis AL, *et al.* Markers of de novo lipogenesis in adipose tissue: associations with small adipocytes and insulin sensitivity in humans. *Diabetologia* 2009;**52**:882–90. doi:10.1007/s00125-009-1300-4

32 Lee JJ, Lambert JE, Hovhannisyan Y, *et al.* Palmitoleic acid is elevated in fatty liver disease and reflects hepatic lipogenesis. *Am J Clin Nutr Nutr* 2015;**101**:34–43. doi:10.3945/ajcn.114.092262

33 Krueger F. Trim galore. A wrapper tool around Cutadapt and FastQC to consistently apply quality and adapter trimming to FastQ files. 2015;:516–7.

34 Xi Y, Li W. BSMAP: whole genome bisulfite sequence MAPping program. *BMC Bioinformatics* 2009;**10**:232. doi:10.1186/1471-2105-10-232

35 Adusumalli S, Mohd Omar MF, Soong R, *et al.* Methodological aspects of whole-genome bisulfite sequencing analysis. *Br Bioinform* 2015;**16**:369–79.

For peer review only

For peer review only - http://bmjopen.bmj.com/site/about/guidelines.xhtml

doi:10.1093/bib/bbu016

36 Ventham NT, Kennedy NA, Adams AT, *et al.* Integrative epigenome-wide analysis demonstrates that DNA methylation may mediate genetic risk in inflammatory bowel disease. *Nat Commun* 2016;**7**:13507. doi:10.1038/ncomms13507

37 Benjamini Y, Hochberg Y. Controlling the false discovery rate: a practical and powerful approach to multiple testing. *J R Stat Soc Ser B*;**1995**:289–300. doi:10.2307/2346101

38 Chatterjee S, Majumder PP. Kalyani cohort – the first platform in Eastern India for longitudinal studies on health and disease parameters in peri-urban setting. *Glob Heal Epidemiol Genom* 2017;**2**:e2. doi:10.1017/gheg.2016.19

39 Nair M, Ali MK, Ajay VS, *et al.* CARRS Surveillance study: design and methods to assess burdens from multiple perspectives. *BMC Public Heal* 2012;**12**:701. doi:10.1186/1471-2458-12-701

For peer review only

For peer review only - http://bmjopen.bmj.com/site/about/guidelines.xhtml

Figure Legends

Figure 1. The de novo lipogenesis pathway

Figure 2. The proposed link between early life determinants and lifestyle exposures on adipose DNL via epigenetic and metabolomics perturbations leading to impaired glucose tolerance.

Figure 3. The overall outline of the InDiMeT study.

Figure 4. Metabolic indicators A. percent of surveyed population with random blood glucose levels >140 mg/dl (measured in 15-54 year old men and 15-49 year old women) and B. percent of surveyed population that are overweight or obese of (i) men and (ii) women in India, Karnataka and the states with minimum and maximum values (adapted from the National Family Health Survey-4).[15]

For peer review only

For peer review only - http://bmjopen.bmj.com/site/about/guidelines.xhtml

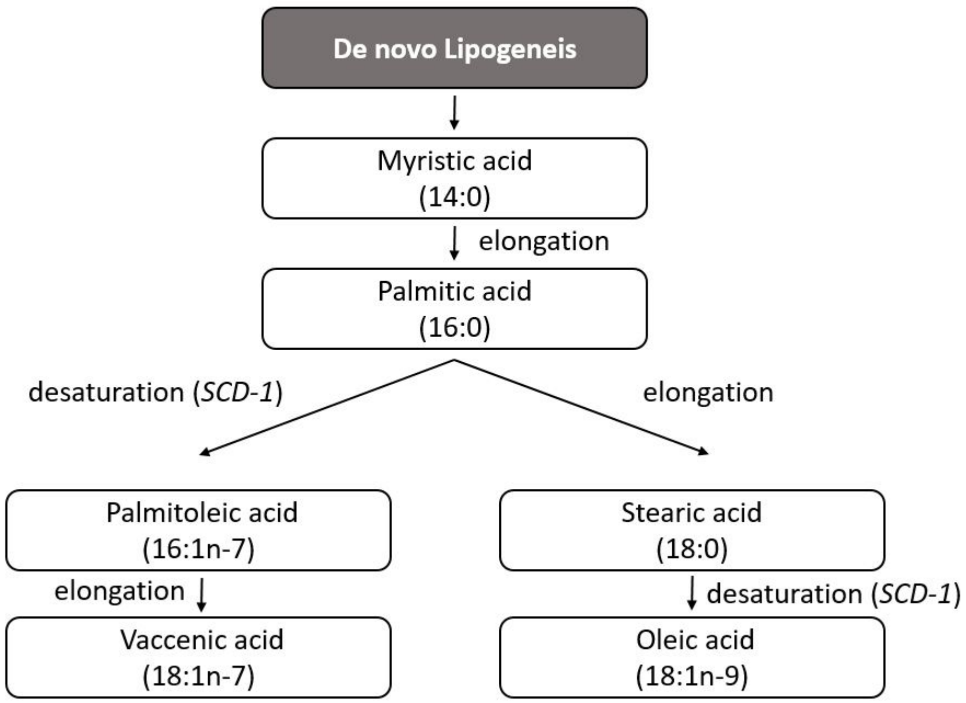

Figure 1. The de novo lipogenesis pathway

19x15mm (1200 x 1200 DPI)

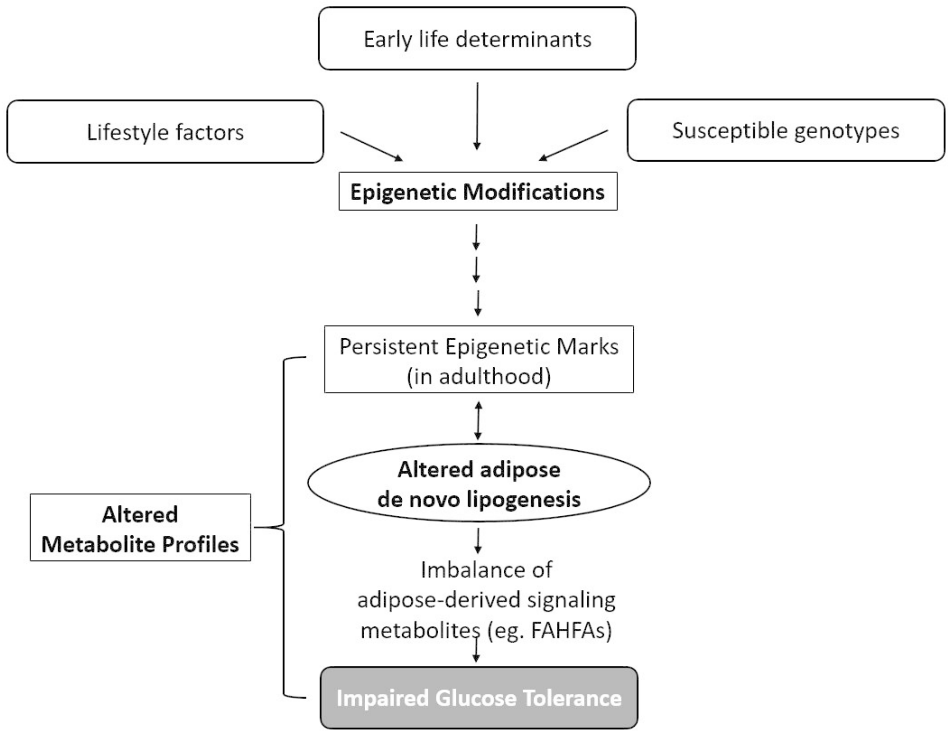

Figure 2. The proposed link between early life determinants and lifestyle exposures on adipose DNL via epigenetic and metabolomics perturbations leading to impaired glucose tolerance.

23x17mm (1200 x 1200 DPI)

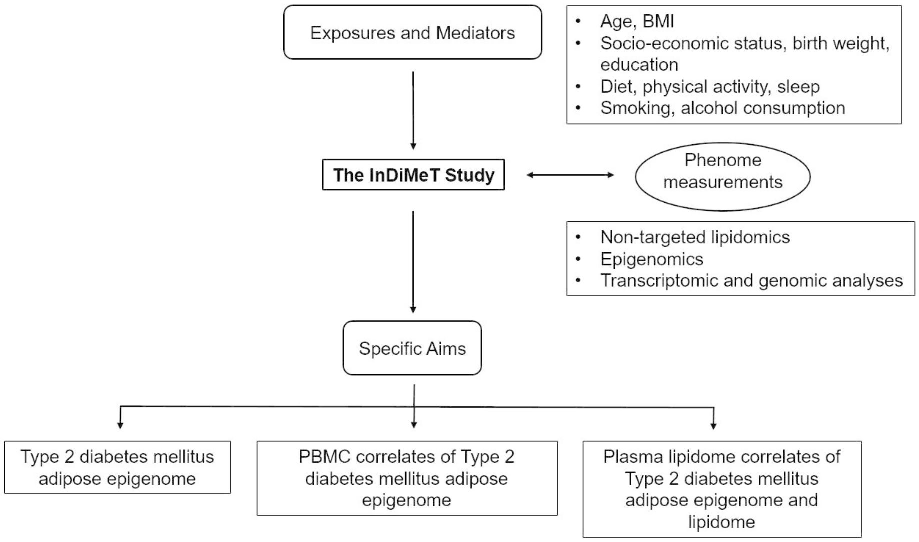

Figure 3. The overall outline of the InDiMeT study.

28x16mm (1200 x 1200 DPI)

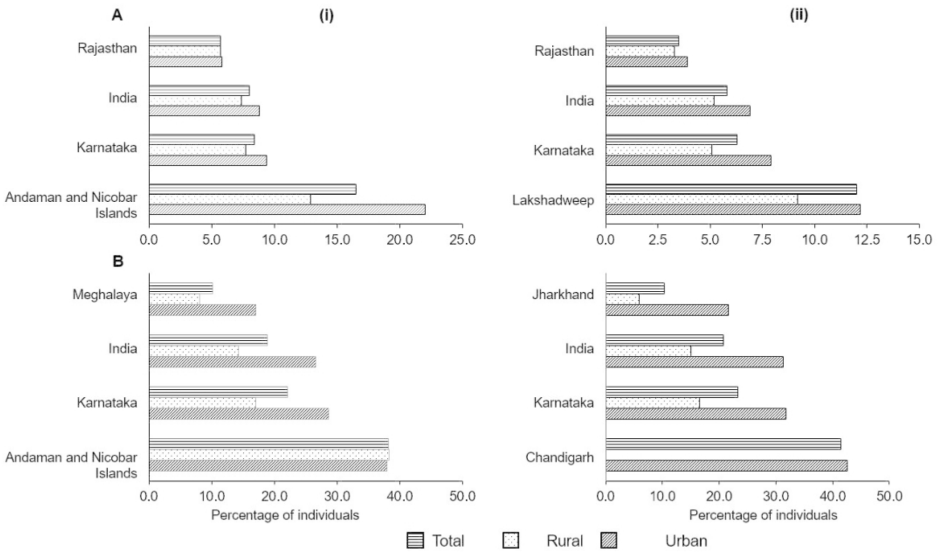

Figure 4. Metabolic indicators A. percent of surveyed population with random blood glucose levels >140 mg/dl (measured in 15-54 year old men and 15-49 year old women) and B. percent of surveyed population that are overweight or obese of (i) men and (ii) women in India, Karnataka and the states with minimum and maximum values (adapted from the National Family Health Survey-4). [18]

29x16mm (1200 x 1200 DPI)

BMJ Open

Protocol for a prospective, observational, deep phenotyping study on adipose epigenetic and lipidomic determinants of metabolic homeostasis in South Asian Indians: The Indian Diabetes and Metabolic Health (InDiMeT) Study

Journal:	*BMJ Open*
Manuscript ID	bmjopen-2020-043644.R2
Article Type:	Protocol
Date Submitted by the Author:	15-Mar-2021
Complete List of Authors:	Nadiger, Nikhil; St John's National Academy of Health Sciences, Division of Nutrition<br>Devi, Sarita; St John's National Academy of Health Sciences, Division of Nutrition<br>Thomas, Tinku; St John's Medical College Hospital, Division of Epidemiology and Biostatistics<br>Sivadas, Ambily; St John's National Academy of Health Sciences, Division of Nutrition<br>Raj-Kuriyan, Rebecca; St John's National Academy of Health Sciences, Division of Nutrition<br>Govindaraj, Sridar; St John's Medical College Hospital, General Surgery<br>Kurpad, Anura; St John's National Academy of Health Sciences, Division of Nutrition<br>mukhopadhyay, arpita; St John's National Academy of Health Sciences, Division of Nutrition
**Primary Subject Heading**:	Diabetes and endocrinology
Secondary Subject Heading:	Diabetes and endocrinology, Genetics and genomics
Keywords:	DIABETES & ENDOCRINOLOGY, Physiology < NATURAL SCIENCE DISCIPLINES, MOLECULAR BIOLOGY, Biochemistry < NATURAL SCIENCE DISCIPLINES, GENETICS

SCHOLARONE™
Manuscripts

I, the Submitting Author has the right to grant and does grant on behalf of all authors of the Work (as defined in the below author licence), an exclusive licence and/or a non-exclusive licence for contributions from authors who are: i) UK Crown employees; ii) where BMJ has agreed a CC-BY licence shall apply, and/or iii) in accordance with the terms applicable for US Federal Government officers or employees acting as part of their official duties; on a worldwide, perpetual, irrevocable, royalty-free basis to BMJ Publishing Group Ltd ("BMJ") its licensees and where the relevant Journal is co-owned by BMJ to the co-owners of the Journal, to publish the Work in this journal and any other BMJ products and to exploit all rights, as set out in our licence.

The Submitting Author accepts and understands that any supply made under these terms is made by BMJ to the Submitting Author unless you are acting as an employee on behalf of your employer or a postgraduate student of an affiliated institution which is paying any applicable article publishing charge ("APC") for Open Access articles. Where the Submitting Author wishes to make the Work available on an Open Access basis (and intends to pay the relevant APC), the terms of reuse of such Open Access shall be governed by a Creative Commons licence – details of these licences and which Creative Commons licence will apply to this Work are set out in our licence referred to above.

Other than as permitted in any relevant BMJ Author's Self Archiving Policies, I confirm this Work has not been accepted for publication elsewhere, is not being considered for publication elsewhere and does not duplicate material already published. I confirm all authors consent to publication of this Work and authorise the granting of this licence.

For peer review only

Protocol for a prospective, observational, deep phenotyping study on adipose epigenetic and lipidomic determinants of metabolic homeostasis in South Asian Indians: The Indian Diabetes and Metabolic Health (InDiMeT) Study

Nikhil Nadiger[1], Sarita Devi[1], Tinku Thomas[2], Ambily Sivadas[1], Rebecca Raj-Kuriyan[1], Sridar Govindaraj[3], Anura V. Kurpad[1] and Arpita Mukhopadhyay[1, *]

[1]Division of Nutrition, St. John's Research Institute, Bangalore, India

[2]Department of Biostatistics, St. John's Medical College and Hospital, Bangalore, India

[3]Department of General Surgery, St. John's Medical College and Hospital, Bangalore, India.

Corresponding author:

Division of Nutrition,

St. John's Research Institute,

Sarjapur Road, Bangalore, India. PIN: 560034

Phone: +91-80-49467000

Fax: +91-80-25501088

Email address: arpitam@sjri.res.in

Word count: 3855

For peer review only

ABSTRACT

Introduction

We describe the rationale and broad study design of the InDiMeT (Indian Diabetes and Metabolic Health) study, a new prospective, observational study incorporating extensive epigenetic (DNA methylation) and lipidomic signatures to examine their association with the dysregulation of adipose de novo lipogenesis (DNL) in South Asian Indians. The InDiMeT study aims to use a case-control design to identify genetic and modifiable-environmental-lifestyle associated determinants of a) epigenomic (DNA methylome) dysregulation of adipose DNL in type 2 diabetes mellitus (T2DM) adipose tissue, b) identify correlates of epigenomic (DNA methylome) dysregulation of adipose DNL in peripheral blood mononuclear cells (PBMCs) from T2DM subjects and c) elucidate plasma lipidomic correlates of adipose DNL in T2DM that can be used as biomarkers of adipose tissue dysfunction.

Methods and analysis

The InDiMeT study will involve recruitment of 176 normoglycaemic and T2DM individuals who will be undergoing laparoscopic surgery for clinical conditions. Extensive phenotyping of the subjects will be conducted and DNA methylome and lipidomic measurements will be made. The adipose DNL pathway genes are likely to be hypermethylated in T2DM patients with corresponding reduction of gene expression. Correlates of epigenomic (DNA methylome) dysregulation of adipose DNL pathway in PBMCs and their adipose and plasma lipidomic signatures in T2DM subjects could act as early markers of development of T2DM.

Ethics and Dissemination

For the InDiMeT study, ethical approval for addressing the specific aims has been obtained from the Institutional Ethics Committee, St. John's Medical College and Hospital, St. John's National Academy of Health Sciences, Bangalore. Findings from this study will be disseminated through scientific publications in peer-reviewed journals, research conferences

For peer review only

For peer review only - http://bmjopen.bmj.com/site/about/guidelines.xhtml

and via presentations to stakeholders, patients, clinicians, public and policymakers through appropriate channels.

Keywords: type 2 diabetes mellitus, South Asian Indians, genotyping, epigenetics, DNA methylome, lipidome, pathophysiology, adipose tissue, de novo lipogenesis

For peer review only

For peer review only - http://bmjopen.bmj.com/site/about/guidelines.xhtml

ARTICLE SUMMARY

Strengths and limitations of this study

- The InDiMeT study will assess the epigenome (DNA methylome)-lipidome connection in relation to their contribution to adipose de novo lipogenesis (DNL) and metabolic homeostasis.
- The prospective case-control study design will permit us to assess the blood epigenetic (DNA methylation) and lipidomic markers that exhibit correlations with adipose DNL dysregulation and can also potentially serve as minimally invasive early biomarkers of dysglycemia.
- Understanding the epigenetic (DNA methylation) mechanisms and their associated lipidomic profiles related to dysregulated adipose DNL in South Asian Indians will provide the rationale for effective interventional strategies targeted to this population and discriminative diagnostic/prognostic biomarkers for prevention of T2DM in this high-risk population.
- Though the sample size of 176 individuals will be sufficient to detect differences in adipose DNA methylation between T2DM and normoglycaemic control subjects, the primary specific aim of this study, it is unlikely to provide required sensitivity to detect dysregulation of adipose DNA methylation in specific sub-groups of T2DM, such as smokers or those with chronic kidney disease.
- The subjects being recruited are the individuals who come for treatment of their preexisting clinical conditions and could not be a random representation of the total population.

INTRODUCTION

For peer review only

For peer review only - http://bmjopen.bmj.com/site/about/guidelines.xhtml

India is experiencing a type 2 diabetes mellitus (T2DM) epidemic, attributed to rapid sociodemographic and nutritional changes.[1] Younger, non-obese South Asian Indians develop insulin resistance, attributed to inappropriately elevated fat accretion.[2] An important reason for elevated/ectopic fat deposition in South Asian Indians may be their uniquely high carbohydrate diets (~65 energy%), which has been associated with elevated risk of T2DM, likely due to induction of hepatic insulin resistance through increased hepatic de novo lipogenesis (DNL, **Figure 1**).[3–5]

Unlike hepatic DNL, adipose DNL is understood to be associated with preservation of insulin sensitivity and an overall favourable metabolic phenotype (‘metabotype’).[6] Strawford et al reported negative correlations between adipose DNL and plasma glucose in humans.[7] Mice overexpressing adipose-specific GLUT4 are obese but paradoxically insulin sensitive, likely mediated by elevated adipocyte glucose influx-induced DNL.[8] Further, adipose-specific GLUT4 overexpression can rescue muscle-specific GLUT4 knockout-mediated insulin resistance, in a background of increased adiposity.[9] Therefore, it can be posited that white adipose tissue (AT) versus hepatic DNL may have opposed effects with respect to obesity-associated metabolic complications. Thus, understanding the contribution of adipose DNL in healthy versus T2DM-associated obesity is critical.

Understanding the epigenetic (DNA methylation) mechanisms and their associated lipidome profiles related to exacerbated adipose DNL in South Asian Indians will further provide the rationale for effective interventional strategies targeted to this population and discriminative diagnostic/prognostic biomarkers for prevention of T2DM in this high-risk population.

Rationale and Specific Aims

For peer review only - http://bmjopen.bmj.com/site/about/guidelines.xhtml

The overarching goal of the Indian Diabetes and Metabolic Health (InDiMeT) study is to identify etiologically valuable determinants that affect susceptibility to type 2 diabetes at individual, sub-group and population level by testing the hypothesis that the epigenetic (DNA methylation) dysregulation of adipose DNL induced by high carbohydrate diet and environmental factors is an important step in the development adipose tissue dysfunction leading to type 2 diabetes (**Figure 2**) in India. The InDiMeT study proposes to achieve this by conducting detailed phenotyping and phenome measurements in a prospective manner in a high-risk population setting. Though animal studies have underlined the epigenetic changes in the adipose associated, few have investigated adipose epigenetics in humans and none in the context of adipose DNL dysregulation and T2DM [10–12] which will help to identify epigenomic dysregulation of adipose DNL in T2DM AT. Although DNA methylation signatures are specific to tissue and cell types, only ~38% of the DNA methylation probes have been reported to be differentially methylated in blood in a tissue-specific fashion, implying likelihood of the rest ~62% probes being conservatively methylated between blood and other tissues.[13] Since blood is an easily available tissue, correlates of epigenomic (DNA methylome) dysregulation of adipose DNL in peripheral blood mononuclear cells (PBMCs) from T2DM subjects will be evaluated to validate the potential use of PBMCs as surrogates of AT dysfunction.

The overall outline of the InDiMeT study is depicted in **Figure 3**. The specific aims of this study are:

1. To identify epigenomic (DNA methylome) dysregulation of adipose de novo lipogenesis (DNL) in T2DM adipose tissue
2. To identify correlates of epigenomic (DNA methylome) dysregulation of adipose DNL in PBMCs from T2DM subjects

For peer review only - http://bmjopen.bmj.com/site/about/guidelines.xhtml

3. To elucidate plasma lipidomic correlates of adipose DNL in T2DM that can be used as biomarkers of adipose tissue dysfunction

METHODS AND ANALYSIS

Study setting

The InDiMeT study is being conducted at St. John's Medical College and Hospital, a tertiary care hospital in Bangalore district, India. It is surrounded by the state of Tamil Nadu in the southeast, the Bangalore Rural district in the northeast and northwest, and the Ramanagara district in the southwest.[14] As per the Indian 2011 Census, the Bangalore district recorded a decadal growth rate increase of 12% in the decade of 2001-2011 compared to the previous decade. This growth was ~4 times higher in the urban areas compared to the rural ones. The sex ratio of the Bangalore district was 916, an 8-point increase compared to the 2001 Census. The literacy rate was 88% while the work participation rate was 44%, higher for males at 62% compared to females at 25%. As per the National Family Health Survey-4, one-third of the women and 26% of the men in Bangalore (Bangalore urban district) are overweight or obese.[15] In Bangalore urban district, prevalence of elevated random blood sugar measurements stands at 8.3% amongst women and 10.9% amongst men which compares to Karnataka state-wide figures of 6.3% for women and 8.4% for men respectively (**Figure 4**).

Study design and population

The InDiMeT study has been designed as a prospective tertiary care hospital-based cohort study. 176 normoglycemic and T2DM individuals will be recruited into the study after explanation of details of the study and obtaining informed consent at the department of General surgery, St. John's Medical College and Hospital, St. John's National Academy of Health Sciences, Bangalore. Dissemination of information about the broader study goals and likely benefits to the community as well as engagement of potential subjects from the hospital was

For peer review only - http://bmjopen.bmj.com/site/about/guidelines.xhtml

initiated in August 2020. Recruitment of subjects commenced from November 19th 2020, after ethical approval for the study had been obtained from the Institutional Ethics Committee. Recruitment of subjects will continue till the recruitment target (176 individuals) is achieved. Strategies elaborated below are being employed for recruitment:

- Chart reviews – From the existing group of patients coming in to the Department of General Surgery, St. John's Medical College and Hospital, subjects are screened and then contacted to request for their participation.
- Databases – From the database of patients coming to the St. John's Medical College and Hospital, those who are eligible to participate in the study are contacted for possible recruitment.

Inclusion criteria: 35-60 years age; normoglycaemic and T2DM; normal and high BMI; men and women who are undergoing laparoscopic surgeries for their clinical conditions such as cholecystectomies, hernia or splenectomy are invited to participate in the study.

Exclusion criteria: Age outside the range of 35-60 years, individuals who are unwilling to participate in the study, individuals participating in any other study and those who tested positive for hepatitis (HBsAg), HIV or syphilis (VDRL) infections. Those who have serious pre-existing medical condition, those undergoing laparoscopic surgery for clinical conditions other than cholecystectomies, hernia or splenectomy will be excluded, and these will be defined as conditions that require chronic or daily medical therapy, including medications, for T2DM, connective tissue diseases, inflammatory bowel disease, active tuberculosis and symptomatic heart disease.

Sample size calculation: Based on the study by Nilsson et al, the sample size required to observe a mean difference of 7% in DNA methylation in AT with an SD of 10% between T2DM and normoglycaemic controls with 90% power and 5% level of significance is 44 per group. As AT DNA methylation differences could be associated with BMI of the subjects, 44

For peer review only - http://bmjopen.bmj.com/site/about/guidelines.xhtml

individuals will be recruited in the normal (BMI<25 kg/m^2) and high BMI (BMI≥25 kg/m^2) groups of normoglycaemic controls and T2DM (35-60 years, men and women).[10]

Findings of this study could be influenced by selection bias in recruitment of the study subjects. Known sources of selection bias, such as geographical location of the study, which influences wide ranging subject parameters such as dietary diversity, levels of sunlight and air pollutant exposures, as well as recruitment of subjects from a hospital, and not from the community, are being addressed by ensuring recruitment of case and control subjects in tandem from a single study site.

Data collection

Socio-demographic data collection

Detailed socio-demographic data will be collected by trained personnel using well-structured questionnaires as elaborated in **Table 1**. The questionnaire will be provided and explained in English or local language in which the subject is most comfortable.

Socioeconomic status will be calculated based on scoring relevant for the Indian scenario.[16]

Table 1. Exposure and outcome measurements within the InDiMeT Study.

General questionnaires
Socio-demographic
Age, ethnicity and religion, family composition, education, employment status and type, household income, socioeconomic status
Medical history
Medical history related to birth weight, chronic and infectious diseases, last one month's morbidity conditions, current and past usage of tobacco (cigarettes, beedis or

For peer review only - http://bmjopen.bmj.com/site/about/guidelines.xhtml

chewing tobacco) and alcohol, family history of diabetes and cardiovascular disease, details on most recent routine clinical investigations (haemoglobin, fasting/random/post-prandial glucose, OGTT glucose, HbA1C, serum insulin and C-peptide, urea, creatinine, Calcium, Phosphorus, TSH, Uric acid, serum cholesterol, HDL, LDL, VLDL, triglycerides, serum amylase and lipase, total protein, albumin, total bilirubin, direct and indirect bilirubin, SGOT, SGPT, alkaline phosphatase, GGT, sodium, potassium, chloride, routine urine test, urine albumin)

Medication and supplement use

Type, brand, dose and duration of medication, vitamin and mineral supplements (clinician prescribed or over the counter)

Dietary habits

3 months food-frequency questionnaire

Physical activity

Daily, weekly and monthly frequency of sports activities, manual labour related hobbies and household chores, sedentary activities; occupational classification in terms of physical activity; sleep habits

Anthropometry, body composition measurements and laboratory assessments

Vital sign measurements

Blood pressure, heart rate and pulse

Body composition and anthropometry measurements

DXA scanning, measurement of height and weight

Laboratory assessments

Blood investigations: Blood glucose level, Glycosylated Haemoglobin (HbA1C), serum insulin, C-peptide, C Reactive Protein (CRP), Glucagon-like peptide (GLP) and Glucose-dependent insulinotropic polypeptide (GIP)

For peer review only - http://bmjopen.bmj.com/site/about/guidelines.xhtml

For peer review only

**Biobanking**
Collection of blood samples
Separation and storage of plasma for lipidomic analyses
Collection of abdominal visceral adipose tissue
Done during laparoscopic surgeries; collection and storage of adipose tissue for lipidomic, epigenomic, genomic and gene expression analyses
**Phenome measurements**
Non-targeted lipidomic analyses
on adipose tissue and plasma samples
Epigenomic analysis
on adipose tissue samples and PBMCs
Gene expression analysis
on adipose tissue samples
Genomic analysis
on PBMCs

OGTT: oral glucose tolerance test, TSH: thyroid-stimulating hormone, HDL: high-density lipoprotein, LDL: low-density lipoprotein, VLDL: very low-density lipoprotein, SGOT: serum glutamic oxaloacetic transaminase, SGPT: serum glutamic pyruvic transaminase, GGT: gamma-glutamyl transferase, DXA: dual-energy X-ray absorptiometry, GAD: glutamic acid decarboxylase, PBMC: peripheral blood mononuclear cells

Medical history and medication and supplement use data collection

Participants will be asked regarding their birth weight, recent morbidity conditions and the medications and supplements they are currently taking. Details of the type and usage of these medications and supplements will be recorded by trained staff (Table 1). Participants will

For peer review only

be requested to provide these details from the prescriptions or their hospital records will be sought after.

Vital sign measurements

Participants will be requested to relax and then blood pressure, heart rate and pulse will be measured and recorded by trained staff.

Body composition measurement by DXA scanning and anthropometry

Participants will be weighed in minimal clothing using a digital scale, to a precision of 0.1 kg. The height of the subjects will be recorded to the nearest 0.1 cm. Whole body and regional body composition will also be estimated using DXA (DPXMD 7254, Lunar Corporation, Madison, WI). The total body fat (BF) will be measured and both fat and muscle (ALST) will be expressed as a percentage of body weight (% BF and % ALST).

Laboratory assessments

Blood samples before and after their surgery will be collected from the participants. The blood sample collected before surgery will be a fasting sample as the participants abstain from eating anything 10 hours before surgery, as a pre-operative requirement for surgery. These samples will be utilized for assessments as elaborated in **Table 1**. Muscle insulin sensitivity and β-cell function will be assessed by the homeostasis model assessment-of insulin resistance (HOMA-IR) and of β-cell function (HOMA-B) respectively.[17,18]

Lifestyle factors

Dietary data collection

For peer review only

For peer review only - http://bmjopen.bmj.com/site/about/guidelines.xhtml

The dietary intake will be assessed using a validated food frequency questionnaire (FFQ). The FFQ will be administered by trained personnel to obtain information about the habitual dietary intake for the preceding 3 months.[19] The nutrients and food groups will be estimated for all the foods listed in the FFQ and summed to obtain the total nutrient or food group intake per day for an individual. Nutrient information will be assessed for 27 macronutrients and micronutrients.

Physical activity data collection

The habitual physical activity and sleep habits of the subjects will be assessed by detailed and validated physical activity questionnaire.[20]

Biobanking

Blood collection and storage

Blood samples will be collected before and after the surgery by phlebotomy in EDTA/serum tubes, kept on ice, and immediately processed for aliquoting and storage at -80°C.

Abdominal visceral adipose tissue collection and storage

Intraabdominal omental adipose tissue will be collected during laparoscopic surgery with clinical indications of cholecystectomy, meshplasty (hernia) or splenectomy. The collected tissue will be appropriately stored for phenome measurements (epigenomic and non-targeted lipidomic analyses) as described below.

Phenome measurements

Epigenomic (DNA methylome) analysis

For peer review only - http://bmjopen.bmj.com/site/about/guidelines.xhtml

For peer review only

Nucleic acids will be extracted from peripheral blood mononuclear cells (PBMCs) and adipose tissue. Epigenome-wide assessment of DNA methylation levels in CpG islands will be done by reduced representation bisulfite sequencing (RRBS) after bisulfite conversion.[10,21,22]

Gene expression by quantitative real-time PCR (qPCR) will be performed with SYBR Green chemistry for the top ten differentially methylated loci and for the adipose DNL pathway related genes: *MLXIPL, SREBP1, ACLY, ACC1, FASN, CPT1A, CPT1B, SCD* and *ELOVL6*. While lysis of citrate to acetyl CoA, the initial substrate of lipogenesis, is catalyzed by ATP-citrate lyase (ACLY) in the cytosol, cytosolic acetyl-CoA carboxylase (ACC1) condenses acetyl CoA into malonyl CoA.[23] Fatty acid synthase (FASN) then sequentially condenses 2-carbon units from malonyl CoA to form myristic acid and palmitic acid. Palmitic acid can then either undergo further elongation by elongase 6 (ELOVL6) or desaturation by stearoyl-CoA desaturate (SCD). Carnitine palmitoyltransferase 1A and 1B (CPT1A and CPT1B), SREBP1 and MLXIPL are involved in regulation of the DNL pathway. CPT1A and CPT1B are required for transport of long-chain fatty acyl-CoAs from cytosol to mitochondria for the beta-oxidation pathway. MLX Interacting Protein Like (MLXIPL), also known as Carbohydrate-Responsive Element-Binding Protein (CHREBP) primarily and Sterol Regulatory Element Binding Transcription Factor 1 (SREBP1), to a lesser extent, control and activate transcription of multiple DNL genes such as ACC1 and ELOVL6 in adipocytes.[24]

Genotyping of the subjects will be done using Metabochip (a custom genotyping array for metabolic and cardiovascular traits) or equivalent single nucleotide polymorphism (SNP) array, such as the Infinium Global Screening Array (Illumina Inc., USA).[25,26]

Non-targeted lipidomic analysis

For peer review only - http://bmjopen.bmj.com/site/about/guidelines.xhtml

For peer review only

Plasma and omental AT samples will be used for non-targeted lipidomic analysis. Samples will be spiked with SPLASH™ Lipidomix Mass Spec standard (Avanti Polar Lipids, USA) before lipid extraction and reconstitution. Non-targeted lipidomic analysis will be performed on a high-resolution analytical platform consisting a Vanquish Flex Binary UHPLC coupled to a Q Exactive Orbitrap, high-resolution accurate-mass spectrometer (LC-HRAM-MS, Thermo Scientific, USA). High resolution accurate mass data generated by Thermo Scientific Q Exactive Orbitrap will be identified and quantified by Thermo Scientific Lipid Search™ software. The following lipid species are of special interest: palmitic acid, stearic acid, oleic acid, myristic acid, palmitoleic acid, acyl carnitines, palmitic acid esters of hydroxyl stearic acids (PAHSAs), furan fatty acid metabolite 3-carboxy-4-methyl-5-propyl-2-furanpropanoic acid (CMPF).[27–30]

Assessment of adipose DNL will be done by determining AT triglyceride (TG) content of the fatty acids myristic acid (14:0), palmitic acid (16:0) and stearic acid (18:0) that have been reported to be correlated with plasma non-esterified fatty acids (NEFA) 14:0 and 18:0 but not with plasma NEFA 15:0, that is exclusively derived from dietary dairy sources.[31] Further, AT lipid species (from non-targeted lipidomic data) that positively correlate with transcript abundances of adipose DNL pathway genes will be identified as indicators of adipose DNL. It should be noted here that though adipose tissue myristic acid, palmitic acid and steric acid contents are not direct measures of adipose DNL, they represent end products of DNL and have been reported to be correlated to expression of genes involved in adipose DNL.[31]

Plasma NEFA correlates of adipose DNL will be identified in the T2DM and normoglycaemic subjects by correlation analysis of plasma lipidomic data with adipose DNL measures [AT triglyceride (TG) content of the fatty acids myristic acid (14:0), palmitic acid (16:0) and stearic acid (18:0)]. This is a novel strategy for estimating adipose DNL and identifying plasma correlates of adipose DNL as such indirect methods do not exist at present

For peer review only - http://bmjopen.bmj.com/site/about/guidelines.xhtml

and the direct stable isotope based method of measuring DNL primarily measures post-prandial hepatic DNL rates.[32]

Data analysis plan

Continuous data will be presented as either mean ± SD or median (quartile1, quartile 3). The normality of distribution will be examined using the Shapiro-Wilk test and Q-Q plots. Demographic and clinical characteristics will be compared between cases of T2DM and controls by Student's t-test, if normally distributed or Mann-Whitney U test, if non-normally distributed, for continuous variables and by Chi-square test for categorical variables.

For analysis of RRBS data, the read sequences from the RRBS libraries will be adapted/quality trimmed using Trim Galore[33] and aligned to the reference human genome using a wildcard mapper like BSMap.[34] DNA methylation scores (β values) will be calculated from the aligned reads.[35] Differentially methylated position (DMP) analysis will be conducted using limma software while adjusting for cell proportions (for PBMCs) and including age and sex as covariates.[36] Statistical significance will be set at $P<0.05$ following adjustment for multiple testing using the Benjamini Hochberg false discovery rate (FDR).[37] Differentially methylated regions (DMRs) will be defined as three or more contiguous probes within a 2 kb distance, each sharing the same direction of methylation change, each achieving a Benjamini Hochberg FDR corrected $P<0.05$ in DMP analysis. Enrichment analysis will be performed to understand the top significantly deregulated gene ontologies and pathways. Additionally, in-depth analysis of methylation status of the CpG islands in the promoter and coding regions of genes associated with the DNL pathway will be conducted.

Association studies between the CpG methylated regions and clinical traits will be performed using linear mixed-model package pyLMM and significant associations will be determined using Bonferroni corrected p-value cut-offs.

For peer review only - http://bmjopen.bmj.com/site/about/guidelines.xhtml

Gene expression values will be estimated by normalizing target gene expression against expression of housekeeping genes followed by comparison of target gene expression between the groups.

Methylation Quantitative Trait Loci (mQTLs) will be identified by testing for association between methylation levels and genotypes of the samples using FDR correction.

Correlation-based clustering analysis of the lipid profiles will be performed and visualized as heatmaps to evaluate the interrelationships between the lipid species and for identifying signatures that differentiate between T2DM and control samples using supervised and unsupervised approaches. For heatmap analysis, only significantly altered features (significant alterations will be defined) will be analyzed and data will be organized using a Euclidean distance map and complete clustering algorithm. Random forest will be used to assess the classification performance of the lipidomics signatures. The significantly perturbed pathways will be identified based on the differentially regulated lipids. For random forest and pathway impact analyses, data will be filtered using the interquartile range (IQR) function and scaled using auto-scaling, in which values are mean-centered and divided by the standard deviation of each variable. For the random forest analysis, the number of predictors to try for each node will be set to the square root of the total number of variables.

To identify the adipose lipid species that correlate with transcript abundances of genes in the adipose DNL pathway, first correlation analysis (Pearson's correlation, for normally distributed data and Spearman's correlation, for non-normally distributed data) will be conducted. This will be followed by regression analysis, with levels of lipid species exhibiting significant correlations as dependent variable and transcript abundance of individual genes in the adipose DNL pathway as independent variables, adjusting for probable confounders such as BMI and sex, if found to be significantly correlated with the dependent variables.

For peer review only - http://bmjopen.bmj.com/site/about/guidelines.xhtml

All statistical analysis will be processed and analyzed using a combination of SPSS Version 25 (IBM, USA), STATA 14 (StataCorp, USA) and R (R Foundation for Statistical Computing, Vienna).

Patient and public involvement

Patients and general public were not invited to comment on the study design and were not consulted to formulate study outcomes. Results for the InDiMeT study will be communicated to the patients and public in two complementary ways. Data related to individual participants of the study will be shared with them, if deemed beneficial for improving their health status, in consultation with clinician collaborators on this study and specific participant or their primary care providers. This is mandated by our Institutional Ethics Committee as well. The overall study results will be disseminated to participating individuals and public via appropriate forums for public science outreach.

ETHICS AND DISSEMINATION

Ethical considerations and informed consent

Institutional ethical approval for the study has been obtained from the Institutional Ethics Committee (IEC), St. John's Medical College and Hospital, St. John's National Academy of Health Sciences, Bangalore (IEC Study Ref No. 246/2020, IEC Study title: "De novo lipogenesis in adiposity and glycaemic control: Epigenetic mechanisms and associated metabolite profiles"). Subject recruitment has been initiated from November 19th 2020.

The objectives and aims of the study are being explained in subject's preferred local language to interested volunteers who fulfill the inclusion criteria. The Patient Information Sheet contains relevant and specific information about the study, collection and storage of

For peer review only - http://bmjopen.bmj.com/site/about/guidelines.xhtml

352 samples, genetic analysis and safe storage of subject data. A written, informed consent is being
353 obtained from the subjects before the start of the protocol.
354
355 Data storage, management and protection
356 Data and resources generated as part of this study will be managed, stored and shared in line
357 with Institutional, State and National policies. In brief, specifics are as follows:
358 • *Organization*: Conventions for naming and storage organization of data files as well as
359 samples from human subjects (adipose and blood) will be set up, documented and
360 circulated amongst study team members at the beginning of the study.
361 • *Storage*: Data will be stored in a secure manner in three copies in two different media.
362 Back-up copies of data will be accompanied with metadata that describe the stored data
363 in a standard, understandable format. Data storage and protection support will be
364 obtained from the Institutional Information Technology team.
365 • *Physical samples*: Samples from the study subjects will be processed and stored as per
366 the experimental protocols. Samples in excess of the requirements of the study
367 objectives will be stored appropriately as per the Institutional policies as well as policies
368 of the peer-reviewed journals where findings from the study will be submitted for
369 possible publications.
370
371 **Dissemination of data**
372 Sequence data generated from this study will be deposited in GenBank and epigenetic
373 data in Gene Expression Omnibus, while raw and processed lipidomic data will be deposited
374 in appropriate repositories such as Metabolomics Workbench or MetaboLights during the time
375 of publication. Findings from the InDiMeT study will be appropriately communicated in
376 refereed journals and forums for scientific and community science outreach.

For peer review only - http://bmjopen.bmj.com/site/about/guidelines.xhtml

For peer review only

SIGNIFICANCE AND OUTLOOK

The InDiMeT study will provide the base and act as a robust tool for mechanistic interpretations of public health and epidemiological aspects of developing T2DM and its complications in the Indian setting. Though a few large, prospective cohort studies to identify the determinants of T2DM are being conducted in India, to the best of our knowledge, none of them involve stated deep phenotyping or extensive phenome measurements of the participants.[38,39] This study was conceptualized to fill this niche in this country. The carefully monitored and quality-controlled collection and archiving of biological samples, along with availability of anthropometric, socio-demographic, nutritional and medical data on the participants will aid toward planning and implementation of an array of hypothesis-driven mechanistic studies.

The InDiMeT study will not be without limitations. Notable ones will include the low probability of participation by individuals of specific subsets of the population due to the voluntary nature of participation. The subjects being recruited are the individuals who come for treatment of their preexisting clinical conditions could not be a random representation of the total population. Further, the study setting being a tertiary care hospital in a large, urban area in India, a country known for its large regional variations in cuisine and culture-associated dietary habits, composition and amounts of habitual dietary intakes of the study subjects are likely to be heterogenous. These factors could act as confounders and limit our ability to detect differences in DNA methylation and lipid profiles that are of low effect sizes. Detailed recording of these factors as proposed, will however help us in understanding if these factors do act as confounders in our study and allow us and others to improve design of similar studies in future.

For peer review only - http://bmjopen.bmj.com/site/about/guidelines.xhtml

Acknowledgements

The contribution of research fellow Ms. Bajanai Nongkhlaw, research assistant Ms. Beena Bose and nutritionist Mr. J. Jayakumar is gratefully acknowledged.

Authors' contributions

The authors of the manuscript and their individual contributions towards the manuscript are as follows. AM, TT and AVK: designed the research; NN, SD and SG: conduct of the study; AM, TT, AS, RR-K and AVK: analysis of the data, performing statistical analyses and interpretation of the results; AM and AVK: obtained funding; AM: had the idea for and led writing of the manuscript and had primary responsibility for the final content of the manuscript; and all authors critically reviewed the manuscript, read and approved the final version of the manuscript.

Funding

This study is supported by the Wellcome Trust/DBT India Alliance Fellowship awarded to AM [grant number IA/CPHI/19/1/504593] and to AVK [grant number IA/M/14/1/501681] and by the Department of Biotechnology, Government of India [Grant Sanction order BT/PR24342/PFN/20/1314/2017 to AM and AVK].

Competing interests

None declared.

For peer review only

References

1 World Health Organization. Global Health Estimates: Deaths by Cause, Age, Sex and Country, 2000-2012. Geneva: 2014.

2 Misra A, Madhavan M, Vikram NK, *et al.* Simple anthropometric measures identify fasting hyperinsulinemia and clustering of cardiovascular risk factors in Asian Indian adolescents. *Metabolism* 2006;**55**:1569–73. doi:10.1016/j.metabol.2006.06.029

3 Mohan V, Radhika G, Sathya RM, *et al.* Dietary carbohydrates, glycaemic load, food groups and newly detected type 2 diabetes among urban Asian Indian population in Chennai, India (Chennai Urban Rural Epidemiology Study 59). *Br J Nutr* 2009;**102**:1498–506. doi:10.1017/S0007114509990468

4 Acheson KJ, Schutz Y, Bessard T, *et al.* Carbohydrate metabolism and de novo lipogenesis in human obesity. *Am J Clin Nutr* 1987;**45**:78–85.

5 Perry RJ, Samuel VT, Petersen KF, *et al.* The role of hepatic lipids in hepatic insulin resistance and type 2 diabetes. *Nature* 2014;**510**:84–91. doi:10.1038/nature13478

6 Yilmaz M, Claiborn KC, Hotamisligil GS. De Novo Lipogenesis Products and Endogenous Lipokines. *Diabetes* 2016;**65**:1800–7. doi:10.2337/db16-0251

7 Strawford A, Antelo F, Christiansen M, *et al.* Adipose tissue triglyceride turnover, de novo lipogenesis, and cell proliferation in humans measured with 2 H 2 O. *Am J Physiol Metab* 2004;**286**:E577–88. doi:10.1152/ajpendo.00093.2003

8 Shepherd PR, Gnudi L, Tozzo E, *et al.* Adipose cell hyperplasia and enhanced glucose disposal in transgenic mice overexpressing GLUT4 selectively in adipose tissue. *J Biol Chem* 1993;**268**:22243–6. doi:10.1007/s00125-008-1025-9

9 Carvalho E, Kotani K, Peroni OD, *et al.* Adipose-specific overexpression of GLUT4 reverses insulin resistance and diabetes in mice lacking GLUT4 selectively in muscle. *Am J Physiol Endocrinol Metab* 2005;**289**:E551–61. doi:10.1152/ajpendo.00116.2005

For peer review only

For peer review only - http://bmjopen.bmj.com/site/about/guidelines.xhtml

10 Nilsson E, Jansson PA, Perfilyev A, *et al.* Altered DNA Methylation and Differential Expression of Genes Influencing Metabolism and Inflammation in Adipose Tissue From Subjects With Type 2 Diabetes. *Diabetes* 2014;**63**:2962–76. doi:10.2337/db13-1459

11 Orozco LD, Farrell C, Hale C, *et al.* Epigenome-wide association in adipose tissue from the METSIM cohort. *Hum Mol Genet* 2018;**27**:1830–46. doi:10.1093/hmg/ddy093

12 Broholm C, Olsson AH, Perfilyev A, *et al.* Epigenetic programming of adipose-derived stem cells in low birthweight individuals. *Diabetologia* 2016;**59**:2664–73. doi:10.1007/s00125-016-4099-9

13 Lowe R, Slodkowicz G, Goldman N, *et al.* The human blood DNA methylome displays a highly distinctive profile compared with other somatic tissues. *Epigenetics* 2015;**10**:274–81. doi:10.1080/15592294.2014.1003744

14 Register General I. Census of India 2011: provisional population totals-India data sheet. 2011.

15 Indian Institute for Population Sciences. National Family Health Survey - 4. Mumbai: 2017. http://rchiips.org/NFHS/factsheet_NFHS-4.shtml (accessed 12 Aug 2019).

16 Kumar N, Kishore J, Gupta N. Kuppuswamy's socioeconomic scale: Updating income ranges for the year 2012. *Indian J Public Heal* 2012;**56**:103. doi:10.4103/0019-557X.96988

17 Matthews DR, Hosker JP, Rudenski AS, *et al.* Homeostasis model assessment: insulin resistance and beta-cell function from fasting plasma glucose and insulin concentrations in man. *Diabetologia* 1985;**28**:412–9.http://www.ncbi.nlm.nih.gov/pubmed/3899825

18 Wallace TM, Levy JC, Matthews DR. Use and abuse of HOMA modeling. *Diabetes*

For peer review only

For peer review only - http://bmjopen.bmj.com/site/about/guidelines.xhtml

Care 2004;**27**:1487–95.http://www.ncbi.nlm.nih.gov/pubmed/15161807

19 Bharathi AV, Kurpad AV, Thomas T, *et al.* Development of food frequency questionnaires and a nutrient database for the Prospective Urban and Rural Epidemiological (PURE) pilot study in South India: methodological issues. *Asia Pac J Clin Nutr* 2008;**17**:178–85.

20 Bharathi A V, Kuriyan R, Kurpad A V, *et al.* Assessment of physical activity using accelerometry, an activity diary, the heart rate method and the Indian Migration Study questionnaire in South Indian adults. *Public Heal Nutr* 2010;**13**:47. doi:10.1017/S1368980009005850

21 Harris RA, Wang T, Coarfa C, *et al.* Comparison of sequencing-based methods to profile DNA methylation and identification of monoallelic epigenetic modifications. *Nat Biotech* 2010;**28**:1097–105. doi:10.1038/nbt.1682

22 Meissner A. Reduced representation bisulfite sequencing for comparative high-resolution DNA methylation analysis. *Nucleic Acids Res* 2005;**33**:5868–77. doi:10.1093/nar/gki901

23 Strable MS, Ntambi JM. Genetic control of de novo lipogenesis: role in diet-induced obesity. *Crit Rev Biochem Mol Biol* 2010;**45**:199–214. doi:10.3109/10409231003667500

24 Song Z, Xiaoli A, Yang F. Regulation and Metabolic Significance of De Novo Lipogenesis in Adipose Tissues. *Nutrients* 2018;**10**:1383. doi:10.3390/nu10101383

25 Morris AP, Voight BF, Teslovich TM, *et al.* Large-scale association analysis provides insights into the genetic architecture and pathophysiology of type 2 diabetes. *Nat Genet* 2012;**44**:981–90. doi:10.1038/ng.2383

26 Voight BF, Kang HM, Ding J, *et al.* The Metabochip, a Custom Genotyping Array for Genetic Studies of Metabolic, Cardiovascular, and Anthropometric Traits. *PLoS Genet*

For peer review only - http://bmjopen.bmj.com/site/about/guidelines.xhtml

2012;**8**:e1002793. doi:10.1371/journal.pgen.1002793

27 Wenjie M, Wu JHY, Wang Q, *et al.* Prospective association of fatty acids in the de novo lipogenesis pathway with risk of type 2 diabetes: The Cardiovascular Health Study. *Am J Clin Nutr* 2015;**101**:153–63. doi:10.3945/ajcn.114.092601

28 Mihalik SJ, Michaliszyn SF, De Las Heras J, *et al.* Metabolomic profiling of fatty acid and amino acid metabolism in youth with obesity and type 2 diabetes: Evidence for enhanced mitochondrial oxidation. *Diabetes Care* 2012;**35**:605–11. doi:10.2337/DC11-1577

29 Yore MM, Syed I, Moraes-Vieira PM, *et al.* Discovery of a Class of Endogenous Mammalian Lipids with Anti-Diabetic and Anti-inflammatory Effects. *Cell* 2014;**159**:318–32. doi:10.1016/j.cell.2014.09.035

30 Schooneman MG, Vaz FM, Houten SM, *et al.* Acylcarnitines: Reflecting or Inflicting Insulin Resistance? *Diabetes* 2013;**62**:1–8. doi:10.2337/db12-0466

31 Roberts R, Hodson L, Dennis AL, *et al.* Markers of de novo lipogenesis in adipose tissue: associations with small adipocytes and insulin sensitivity in humans. *Diabetologia* 2009;**52**:882–90. doi:10.1007/s00125-009-1300-4

32 Lee JJ, Lambert JE, Hovhannisyan Y, *et al.* Palmitoleic acid is elevated in fatty liver disease and reflects hepatic lipogenesis. *Am J Clin Nutr Nutr* 2015;**101**:34–43. doi:10.3945/ajcn.114.092262

33 Krueger F. Trim galore. A wrapper tool around Cutadapt and FastQC to consistently apply quality and adapter trimming to FastQ files. 2015;:516–7.

34 Xi Y, Li W. BSMAP: whole genome bisulfite sequence MAPping program. *BMC Bioinformatics* 2009;**10**:232. doi:10.1186/1471-2105-10-232

35 Adusumalli S, Mohd Omar MF, Soong R, *et al.* Methodological aspects of whole-genome bisulfite sequencing analysis. *Br Bioinform* 2015;**16**:369–79.

For peer review only

523 doi:10.1093/bib/bbu016

524 36 Ventham NT, Kennedy NA, Adams AT, *et al.* Integrative epigenome-wide analysis
525 demonstrates that DNA methylation may mediate genetic risk in inflammatory bowel
526 disease. *Nat Commun* 2016;**7**:13507. doi:10.1038/ncomms13507

527 37 Benjamini Y, Hochberg Y. Controlling the false discovery rate: a practical and
528 powerful approach to multiple testing. *J R Stat Soc Ser B*;**1995**:289–300.
529 doi:10.2307/2346101

530 38 Chatterjee S, Majumder PP. Kalyani cohort – the first platform in Eastern India for
531 longitudinal studies on health and disease parameters in peri-urban setting. *Glob Heal*
532 *Epidemiol Genom* 2017;**2**:e2. doi:10.1017/gheg.2016.19

533 39 Nair M, Ali MK, Ajay VS, *et al.* CARRS Surveillance study: design and methods to
534 assess burdens from multiple perspectives. *BMC Public Heal* 2012;**12**:701.
535 doi:10.1186/1471-2458-12-701

For peer review only

536 **Figure Legends**

Figure 1. The de novo lipogenesis pathway

Figure 2. The proposed link between early life determinants and lifestyle exposures on adipose DNL via epigenetic and metabolomics perturbations leading to impaired glucose tolerance.

Figure 3. The overall outline of the InDiMeT study.

Figure 4. Metabolic indicators A. percent of surveyed population with random blood glucose levels >140 mg/dl (measured in 15-54 year old men and 15-49 year old women) and B. percent of surveyed population that are overweight or obese of (i) men and (ii) women in India, Karnataka and the states with minimum and maximum values (adapted from the National Family Health Survey-4).[15]

For peer review only

For peer review only - http://bmjopen.bmj.com/site/about/guidelines.xhtml

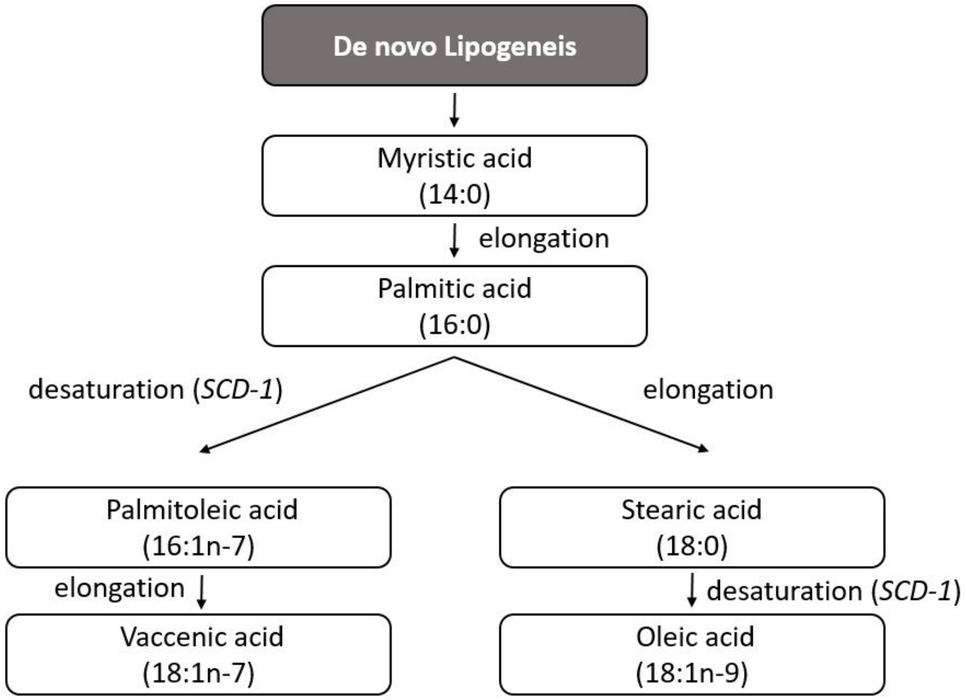

Figure 1. The de novo lipogenesis pathway

19x15mm (1200 x 1200 DPI)

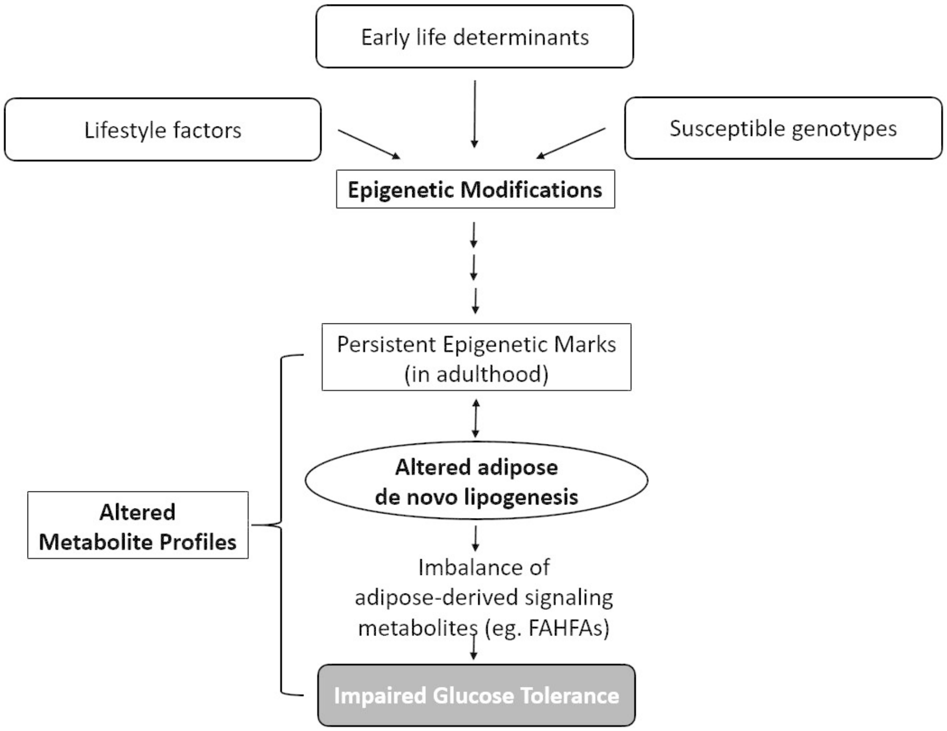

Figure 2. The proposed link between early life determinants and lifestyle exposures on adipose DNL via epigenetic and metabolomics perturbations leading to impaired glucose tolerance.

23x17mm (1200 x 1200 DPI)

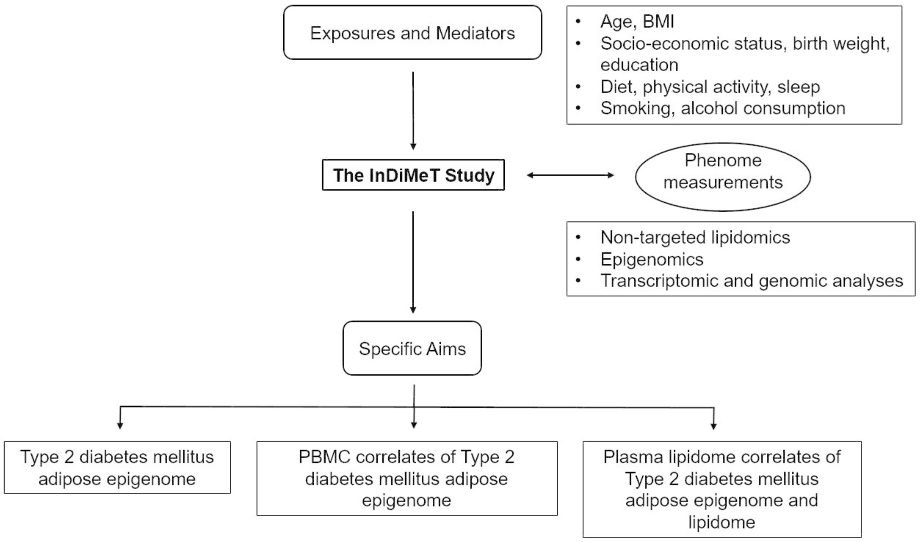

Figure 3. The overall outline of the InDiMeT study.

28x16mm (1200 x 1200 DPI)

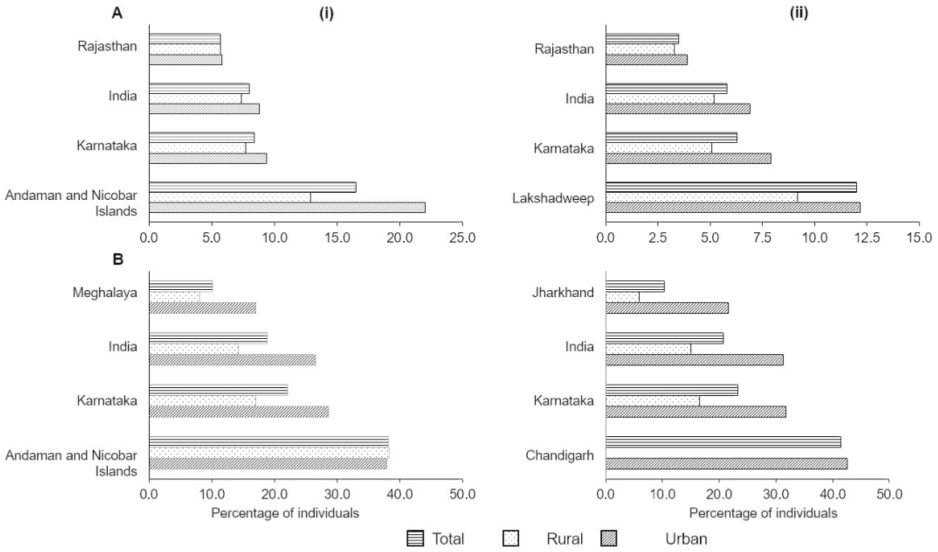

Figure 4. Metabolic indicators A. percent of surveyed population with random blood glucose levels >140 mg/dl (measured in 15-54 year old men and 15-49 year old women) and B. percent of surveyed population that are overweight or obese of (i) men and (ii) women in India, Karnataka and the states with minimum and maximum values (adapted from the National Family Health Survey-4). [18]

29x16mm (1200 x 1200 DPI)

STROBE Statement—Checklist of items that should be included in reports of ***case-control studies***

	Item No.	Recommendation	Page No.
**Title and abstract**	1	(*a*) Indicate the study's design with a commonly used term in the title or the abstract	1, 2
		(*b*) Provide in the abstract an informative and balanced summary of what was done and what was found	2
**Introduction**			
Background/rationale	2	Explain the scientific background and rationale for the investigation being reported	5
Objectives	3	State specific objectives, including any prespecified hypotheses	6, 7
**Methods**			
Study design	4	Present key elements of study design early in the paper	7
Setting	5	Describe the setting, locations, and relevant dates, including periods of recruitment, exposure, follow-up, and data collection	7-13
Participants	6	(*a*) Give the eligibility criteria, and the sources and methods of case ascertainment and control selection. Give the rationale for the choice of cases and controls	8
		(*b*) For matched studies, give matching criteria and the number of controls per case	8
Variables	7	Clearly define all outcomes, exposures, predictors, potential confounders, and effect modifiers. Give diagnostic criteria, if applicable	9-15
Data sources/ measurement	8*	For each variable of interest, give sources of data and details of methods of assessment (measurement). Describe comparability of assessment methods if there is more than one group	9-15
Bias	9	Describe any efforts to address potential sources of bias	9
Study size	10	Explain how the study size was arrived at	8-9
Quantitative variables	11	Explain how quantitative variables were handled in the analyses. If applicable, describe which groupings were chosen and why	16-17
Statistical methods	12	(*a*) Describe all statistical methods, including those used to control for confounding	16-17
		(*b*) Describe any methods used to examine subgroups and interactions	-
		(*c*) Explain how missing data were addressed	-
		(*d*) If applicable, explain how matching of cases and controls was addressed	-
		(*e*) Describe any sensitivity analyses	-
**Results**			
Participants	13*	(a) Report numbers of individuals at each stage of study—eg numbers potentially eligible, examined for eligibility, confirmed eligible, included in the study, completing follow-up, and analysed	-
		(b) Give reasons for non-participation at each stage	-
		(c) Consider use of a flow diagram	-
Descriptive data	14*	(a) Give characteristics of study participants (eg demographic, clinical, social) and information on exposures and potential confounders	-
		(b) Indicate number of participants with missing data for each variable of interest	-
Outcome data	15*	Report numbers in each exposure category, or summary measures of exposure	-
Main results	16	(*a*) Give unadjusted estimates and, if applicable, confounder-adjusted estimates and their precision (eg, 95% confidence interval). Make clear which confounders were adjusted for and why they were included	-
		(*b*) Report category boundaries when continuous variables were categorized	-
		(*c*) If relevant, consider translating estimates of relative risk into absolute risk for a meaningful time period	-

For peer review only

Other analyses	17	Report other analyses done—eg analyses of subgroups and interactions, and sensitivity analyses	-
**Discussion**			
Key results	18	Summarise key results with reference to study objectives	-
Limitations	19	Discuss limitations of the study, taking into account sources of potential bias or imprecision. Discuss both direction and magnitude of any potential bias	4, 20
Interpretation	20	Give a cautious overall interpretation of results considering objectives, limitations, multiplicity of analyses, results from similar studies, and other relevant evidence	-
Generalisability	21	Discuss the generalisability (external validity) of the study results	-
**Other information**			
Funding	22	Give the source of funding and the role of the funders for the present study and, if applicable, for the original study on which the present article is based	21

*Give information separately for cases and controls.

Note: Since this is a protocol for a prospective study, some of the information is not available.

Note: An Explanation and Elaboration article discusses each checklist item and gives methodological background and published examples of transparent reporting. The STROBE checklist is best used in conjunction with this article (freely available on the Web sites of PLoS Medicine at http://www.plosmedicine.org/, Annals of Internal Medicine at http://www.annals.org/, and Epidemiology at http://www.epidem.com/). Information on the STROBE Initiative is available at http://www.strobe-statement.org.

For peer review only
